# Environmental consequences of interacting effects of changes in stratospheric ozone, ultraviolet radiation, and climate: UNEP Environmental Effects Assessment Panel, Update 2024

**DOI:** 10.1007/s43630-025-00687-x

**Published:** 2025-03-17

**Authors:** Patrick J. Neale, Samuel Hylander, Anastazia T. Banaszak, Donat-P. Häder, Kevin C. Rose, Davide Vione, Sten-Åke Wängberg, Marcel A. K. Jansen, Rosa Busquets, Mads P. Sulbæk Andersen, Sasha Madronich, Mark L. Hanson, Tamara Schikowski, Keith R. Solomon, Barbara Sulzberger, Timothy J. Wallington, Anu M. Heikkilä, Krishna K. Pandey, Anthony L. Andrady, Laura S. Bruckman, Christopher C. White, Liping Zhu, Germar H. Bernhard, Alkiviadis Bais, Pieter J. Aucamp, Gabriel Chiodo, Raúl R. Cordero, Irina Petropavlovskikh, Rachel E. Neale, Catherine M. Olsen, Simon Hales, Aparna Lal, Gareth Lingham, Lesley E. Rhodes, Antony R. Young, T. Matthew Robson, Sharon A. Robinson, Paul W. Barnes, Janet F. Bornman, Anna B. Harper, Hanna Lee, Roy Mackenzie Calderón, Rachele Ossola, Nigel D. Paul, Laura E. Revell, Qing-Wei Wang, Richard G. Zepp

**Affiliations:** 1 Environmental Research Center, Smithsonian Institution, Edgewater, MD USA; 2 Centre for Ecology and Evolution in Microbial Model Systems, Linnaeus University, Kalmar, Sweden; 3 Unidad Académica de Sistemas Arrecifales, Universidad Nacional Autónoma de México, Puerto Morelos, Mexico; 4 Biology, Friedrich-Alexander-University (Retired), Erlangen, Germany; 5 Department of Biological Sciences and Department of Civil and Environmental Engineering, Rensselaer Polytechnic Institute, Troy, NY USA; 6 Department of Chemistry, University of Turin, Turin, Italy; 7 Department of Marine Sciences, University of Gothenburg, Gotheburg, Sweden; 8 School of Biological, Earth and Environmental Sciences, Environmental Research Institute, University College Cork, Cork, Ireland; 9 Chemical and Pharmaceutical Sciences, Kingston University London, Kingston Upon Thames, UK; 10 Civil Environmental & Geomatic Engineering, University College London, London, UK; 11 Department of Chemistry and Biochemistry, California State University, Northridge, CA USA; 12 Atmospheric Chemistry Observations and Modeling, National Center for Atmospheric Research, Boulder, CO USA; 13 USDA UV-B Monitoring and Research Program, Colorado State University, Fort. Collins, CO USA; 14 Department of Environment and Geography, University of Manitoba, Winnipeg, MB Canada; 15 Working Group Environmental Epidemiology, IUF-Leibniz Research Institute for Environmental Medicine, Düsseldorf, Germany; 16 Department of Environment and Health, School of Public Health, University of Bielefeld, Bielefeld, Germany; 17 School of Environmental Sciences, University of Guelph, Guelph, ON Canada; 18 Retired From Eawag, Swiss Federal Institute of Aquatic Science and Technology, Dübendorf, Switzerland; 19 Center for Sustainable Systems, School for Environment and Sustainability, University of Michigan, Ann Arbor, MI USA; 20 Climate Research, Finnish Meteorological Institute, Helsinki, Finland; 21 Indian Academy of Wood Science, Bengaluru, India; 22 Chemical and Biomolecular Engineering, North Carolina State University, Raleigh, NC USA; 23 Department of Materials Science and Engineering, Case Western Reserve University, Cleveland, OH USA; 24 Environment and Health, Ramboll Management Consulting, Arlington, VA USA; 25 State Key Laboratory for Modification of Chemical Fibers and Polymer Materials, College of Materials Science and Engineering, Donghua University, Shanghai, China; 26 Biospherical Instruments, Inc., San Diego, CA, USA; 27 Laboratory of Atmospheric Physics, Aristotle University of Thessaloniki, Thessaloniki, Greece; 28 Ptersa Environmental Consultant, Pretoria, South Africa; 29 Institute of Geosciences, Spanish National Research Council (IGEO-UCM-CSIC), Madrid, Spain; 30 Institute for Atmospheric and Climate Science, ETH Zurich, Zurich, Switzerland; 31 Department of Physics, Universidad de Santiago, Santiago, Chile; 32 Cooperative Institute for Research in Environmental Sciences, University of Colorado, Boulder, CO USA; 33 Population Health Program, QIMR Berghofer Medical Research Institute, Brisbane, Australia; 34 School of Public Health, University of Queensland, Brisbane, Australia; 35 Faculty of Medicine, The University of Queensland, Brisbane, Australia; 36 Public Health, University of Otago, Wellington, New Zealand; 37 National Centre for Epidemiology and Population Health, The Australian National University, Canberra, Australia; 38 Centre for Ophthalmology and Visual Science (Incorporating Lion’s Eye Institute), University of Western Australia, Perth, Australia; 39 Centre for Eye Research Ireland, Environmental Sustainability and Health Institute, Technological University Dublin, Dublin, Ireland; 40 School of Biological Sciences, University of Manchester, Manchester, UK; 41 Dermatology Centre, Salford Royal Hospital, Manchester, UK; 42 King’s College London, London, UK; 43 UK National School of Forestry, Institute of Science and Environment, University of Cumbria, Ambleside, UK; 44 Viikki Plant Science Centre, Faculty of Biological and Environmental Sciences, University of Helsinki, Helsinki, Finland; 45 Securing Antarctica’s Environmental Future, University of Wollongong, Wollongong, Australia; 46 Environmental Futures, University of Wollongong, Wollongong, Australia; 47 Department of Biological Sciences and Environment Program, Loyola University, New Orleans, LA USA; 48 Food Futures Institute, Murdoch University, Perth, Australia; 49 Department of Geography, University of Georgia, Athens, GA USA; 50 Department of Biology, Norwegian University of Science and Technology, Trondheim, Norway; 51 Cape Horn International Center, Universidad de Magallanes, Puerto Williams, Chile; 52 Millennium Institute Biodiversity of Antarctic and Subantarctic Ecosystems, Santiago, Chile; 53 Department of Chemistry, Colorado State University, Fort Collins, CO USA; 54 Lancaster Environment Centre, Lancaster University, Lancaster, UK; 55 School of Physical and Chemical Sciences, University of Canterbury, Christchurch, New Zealand; 56 Institute of Applied Ecology, Chinese Academy of Sciences, Shenyang, China; 57 Office of Research and Development, United States Environmental Protection Agency (retired), Athens, GA USA; 58 Department of Chemistry, University of Copenhagen, Copenhagen, Denmark

## Abstract

This Assessment Update by the Environmental Effects Assessment Panel (EEAP) of the United Nations Environment Programme (UNEP) addresses the interacting effects of changes in stratospheric ozone, solar ultraviolet (UV) radiation, and climate on the environment and human health. These include new modelling studies that confirm the benefits of the Montreal Protocol in protecting the stratospheric ozone layer and its role in maintaining a stable climate, both at low and high latitudes. We also provide an update on projected levels of solar UV-radiation during the twenty-first century. Potential environmental consequences of climate intervention scenarios are also briefly discussed, illustrating the large uncertainties of, for example, Stratospheric Aerosol Injection (SAI). Modelling studies predict that, although SAI would cool the Earth’s surface, other climate factors would be affected, including stratospheric ozone depletion and precipitation patterns. The contribution to global warming of replacements for ozone-depleting substances (ODS) are assessed. With respect to the breakdown products of chemicals under the purview of the Montreal Protocol, the risks to ecosystem and human health from the formation of trifluoroacetic acid (TFA) as a degradation product of ODS replacements are currently *de minimis*. UV-radiation and climate change continue to have complex interactive effects on the environment due largely to human activities. UV-radiation, other weathering factors, and microbial action contribute significantly to the breakdown of plastic waste in the environment, and in affecting transport, fate, and toxicity of the plastics in terrestrial and aquatic ecosystems, and the atmosphere. Sustainability demands continue to drive industry innovations to mitigate environmental consequences of the use and disposal of plastic and plastic-containing materials. Terrestrial ecosystems in alpine and polar environments are increasingly being exposed to enhanced UV-radiation due to earlier seasonal snow and ice melt because of climate warming and extended periods of ozone depletion. Solar radiation, including UV-radiation, also contributes to the decomposition of dead plant material, which affects nutrient cycling, carbon storage, emission of greenhouse gases, and soil fertility. In aquatic ecosystems, loss of ice cover is increasing the area of polar oceans exposed to UV-radiation with possible negative effects on phytoplankton productivity. However, modelling studies of Arctic Ocean circulation suggests that phytoplankton are circulating to progressively deeper ocean layers with less UV irradiation. Human health is also modified by climate change and behaviour patterns, resulting in changes in exposure to UV-radiation with harmful or beneficial effects depending on conditions and skin type. For example, incidence of melanoma has been associated with increased air temperature, which affects time spent outdoors and thus exposure to UV-radiation. Overall, implementation of the Montreal Protocol and its Amendments has mitigated the deleterious effects of high levels of UV-radiation and global warming for both environmental and human health.

**Supplementary Information:**

The online version contains supplementary material available at 10.1007/s43630-025-00687-x.

## Changes in the ozone layer and ultraviolet radiation and their interaction with the climate system

Changes in stratospheric ozone concentrations are inversely correlated with changes in solar ultraviolet (UV) radiation at the Earth’s surface. Quantifying this relationship was historically the main focus for evaluating the effects of ozone depletion and ozone recovery on human health and ecosystems. During the last years, it has become increasingly clear that many effects of changes in ozone—as well as the impact of the Montreal Protocol on protecting the ozone layer—are indirect and modulated by ozone–climate interactions. This section emphasises these indirect effects by assessing, among others: the benefit of the Montreal Protocol for preserving tropical ozone and global precipitation patterns; the links between Arctic ozone depletion and recovery on surface climate at lower latitudes; the association between the recently observed cooling of the Southern Ocean and the Antarctic ozone hole; the effect of the 2022 Hunga Tonga–Hunga Ha’apai eruption on ozone, UV-radiation, and climate in context of the Montreal Protocol. In addition, we assess a new projection of UV-radiation during the twenty-first century and its dependence on the future trajectory of greenhouse gas emissions.

### Benefit of the Montreal Protocol for preserving tropical ozone, atmospheric circulation, and precipitation patterns

**Fig. 1:**
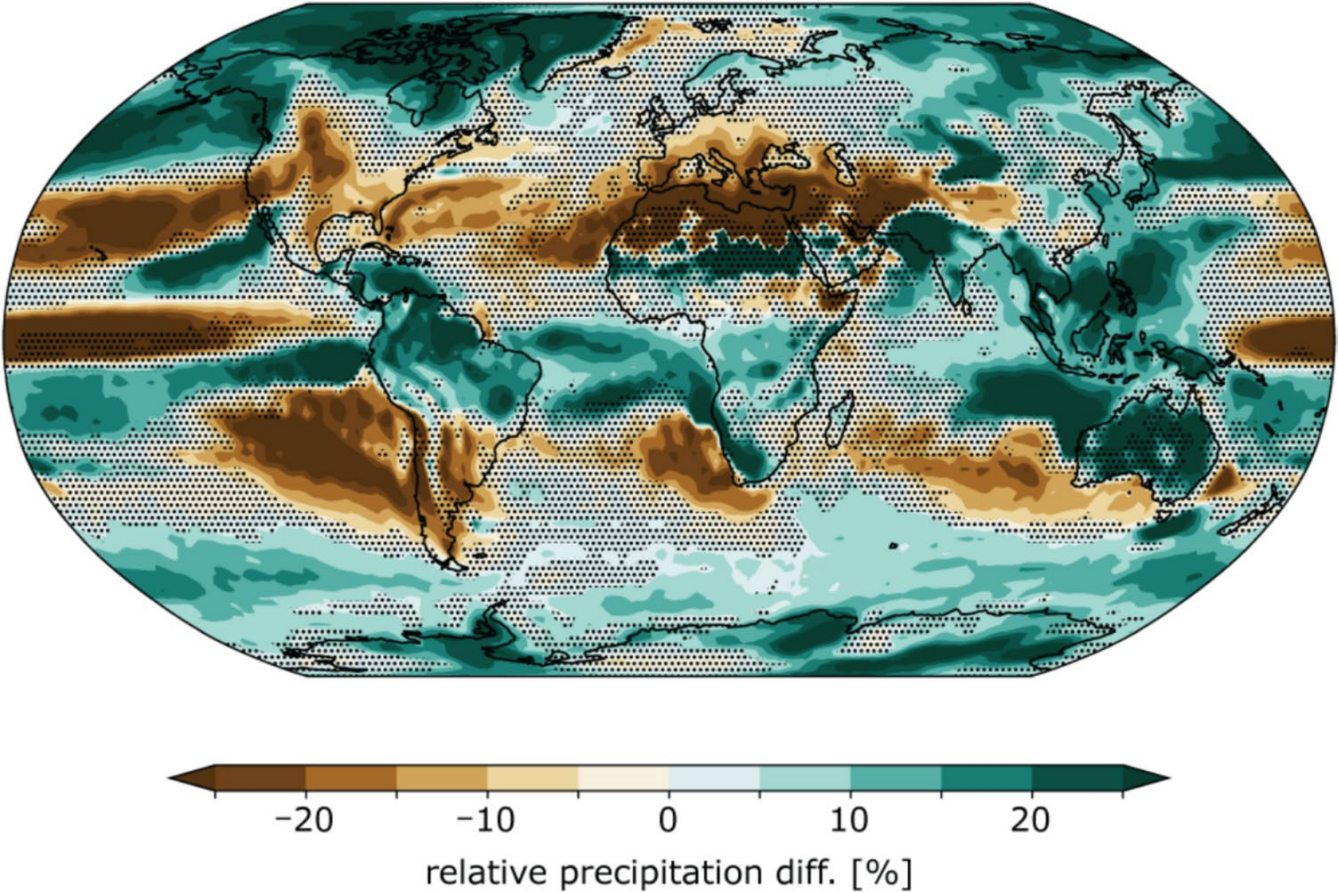
Geographical distribution of the annual-mean difference in precipitation between the “no Montreal Protocol” simulation and a simulation based on the “middle of the road” (SSP2-4.5, see footnote 1) pathway of greenhouse gas emissions, averaged over the period 2090–2100 (in %). Green areas indicate increased precipitation that would have occurred without the Montreal Protocol, whilst brown areas indicate decreases. Areas where the statistical significance of the signal is less than 90% are marked by dots. [4] Reproduced from *Egorova *et al.

Without the Montreal Protocol (i.e., the “world avoided” scenario), emissions of ozone-depleting substances (ODS) would have continued to increase and large decreases in tropical stratospheric ozone would have occurred by the end of the twenty-first century [1, 2]. According to new single-model world-avoided studies based on the SSP2-4.5 scenario [3, 4], these large decreases in tropical ozone would have drastically cooled the tropical tropopause, thereby reducing the equator-to-pole temperature gradient and weakening the winter polar vortices in both hemispheres. This would have also pushed the jet streams and climate zones equatorward in both hemispheres, leading to substantial changes in large-scale circulation and resulting in increased precipitation in the Arctic, the Amazon, Indonesia, India, the Sahel, Australia, the Southern Ocean and coastal areas of Antarctica, and decreased rainfall in the Rocky Mountains, Southern Europe, the Mediterranean, the tropical Pacific Ocean, Chile, Argentina, and the oceans west of Chile, South Africa, and southern Australia (Fig. 1).

On a regional scale, these changes in circulation would have also modulated the direct radiative warming from ODS, which are potent greenhouse gases [5]. According to the new studies [3, 4], continued emissions of ODS would have increased the average global surface temperature by 2.3 °C over the twenty-first century, with the strongest warming by up to 12 °C predicted over parts of the Arctic. These increases are consistent with a study published in 2019 [6] and the conclusions in our previous assessment [7]. The new studies reaffirm the important benefit of the Montreal Protocol for protecting the Earth’s climate.

### Projections of ultraviolet radiation during the twenty-first century

Projections of solar ultraviolet (UV) radiation during the twenty-first century with the Montreal Protocol in force depend strongly on scenarios defining the atmospheric concentrations of greenhouse gases and aerosols. These concentrations determine the evolution of ozone, clouds, and surface reflectivity, which in turn control the amount of UV-radiation at the Earth’s surface. Previous assessments [7, 8] reported changes in UV-radiation during the twenty-first century based on four Representative Concentration Pathway (RCP) scenarios. A new study [9] provided projections of future UV-radiation for cloud-free conditions based on total column ozone and aerosol optical depth data from six CMIP6 (Coupled Model Intercomparison Project Phase 6) models following four Shared Socio-economic Pathway (SSP) scenarios^1^ [10], which supersede the RCP scenarios. The study compared levels of UV-radiation averaged over the periods 2041–2060 and 2081–2100, relative to 1995–2014. For both periods, the largest increases in the UV Index in the Northern Hemisphere are found in June under the SSP1-2.6 and SSP2-4.5 scenarios, which assume a large decrease in air pollutants, resulting in substantial (up to 60%) decreases in aerosol optical depth. By the end of the century, increases in the UV Index between 1.5 and 3.5 are projected for eastern and south-central China, the United States, the Middle East, and India, leading to very high (8–10) and extreme (≥ 11) UV Index values at some places. However, the absolute levels of UV-radiation are not expected to exceed those of the 1950s as reported previously [7, 8]. Instead, the projected increases in UV-radiation mainly reflect a return to more natural levels as air pollution is being reduced. In contrast, changes in UV Index under the SSP3-7.0 scenario, which assumes continued reliance on fossil fuels, indicate decreases in the UV Index of up to 2.5 in June in the Northern Hemisphere. Over the Southern Ocean and Antarctica, the UV Index is expected to decrease in December by up to 1.5 under all SSPs due to the projected recovery of the stratospheric ozone layer over the twenty-first century. Because this study did not consider effects of future changes in clouds due to the warming climate and changes in surface albedo by melting of snow or ice, the projected changes in UV-radiation may be biased (high or low) in areas where clouds and albedo change substantially in the future.

### Links between Arctic ozone and surface climate in the Northern Hemisphere

There is new evidence that strong stratospheric ozone depletion in the Arctic during spring is associated with less precipitation in East Asia. Specifically, observations have shown that a decrease in Arctic stratospheric ozone during March is correlated with decreased precipitation over Eastern China due to changes in atmospheric circulation [11, 12]. However, the spatial extent and magnitude of the observed correlations between precipitation and ozone are currently not fully reproduced by the climate models used in these studies.

### Arctic ozone depletion and UV-radiation during the twenty-first century

Using projections of stratospheric halogen loading with forecasts of stratospheric temperatures derived from general circulation models, von der Gathen [13] concluded that severe episodic Arctic ozone loss could persist or even worsen during the twenty-first century if greenhouse gases in the atmosphere continue to rise. This finding was challenged in a study by Polvani et al. [14] who found, based on chemistry-climate models with interactive ozone chemistry, that Arctic ozone depletion will lessen over this period. These results were disputed by von der Gathen et al. [15] who argued that the projected loss found in their previous study is limited to the polar vortex area, whilst the study by Polvani et al. [14] considers the polar cap (latitudes of 60–90° N) and uses models that have difficulty representing observed total column ozone in the Arctic during spring. This controversy has been partially resolved by a recent analysis [16], which found that polar cap Arctic ozone depletion will diminish over the twenty-first century. This study can be considered reliable, because it is based on several models that reproduce recently observed Arctic ozone minima. Hence, extreme Arctic ozone depletion over an area as extensive as the one observed in 2020 [17] and associated substantial increases in UV-radiation [18] will become less likely in the future as concentrations of ODS are declining.

### Effect of Arctic ozone recovery on surface climate

Aside from variations on inter-annual time-scales, one recent study suggests that long-term future trends in Arctic stratospheric ozone may influence stratospheric circulation and its coupling to surface climate [19]. Specifically, the projected increases in Arctic ozone during springtime would weaken the polar vortex, counteracting the strengthening of the vortex resulting from increasing greenhouse gases. This indicates that increases in Arctic ozone may lead to higher surface pressure over the Arctic, resulting in more frequent cloudless conditions. The net effect of increasing Arctic ozone and decreasing cloudiness on UV-radiation is still unknown.

### Link between the cooling of the Southern Ocean and the Antarctic ozone hole

There have been recent suggestions that the Antarctic ozone hole may affect sea surface temperature patterns over the Southern Ocean, and in particular, the recent cooling trend between 1979 and 2021 [20]. The link was established by correlating the Southern Annular Mode (SAM^2^) index with anomalies in sea surface temperature. A study using both observations and climate modelling over a similar period (1979–2022) reassessed this hypothesis by analysing the observed multi-decadal cooling of the Southern Ocean [21]. Positive SAM anomalies were found to induce cooling of the sea surface, but this seasonal-to-inter-annual modulation contributed only minimally to the long-term cooling trend. Hence, the SAM trend in recent decades, which has been shown to be influenced by the ozone hole [22], is unlikely to be the main cause of the observed cooling of the Southern Ocean, pointing to a limited role of the Antarctic ozone hole. These findings indicate that global climate models capture the inter-annual relationship between SAM and sea surface temperature relationship well, but generally fail to simulate the observed multi-decadal cooling, highlighting the complexity of the interactions between ozone, climate, and trends in sea surface temperatures.

### Effect of the Hunga Tonga–Hunga Ha’apai eruption on ozone, UV-radiation, and climate

**Fig. 2:**
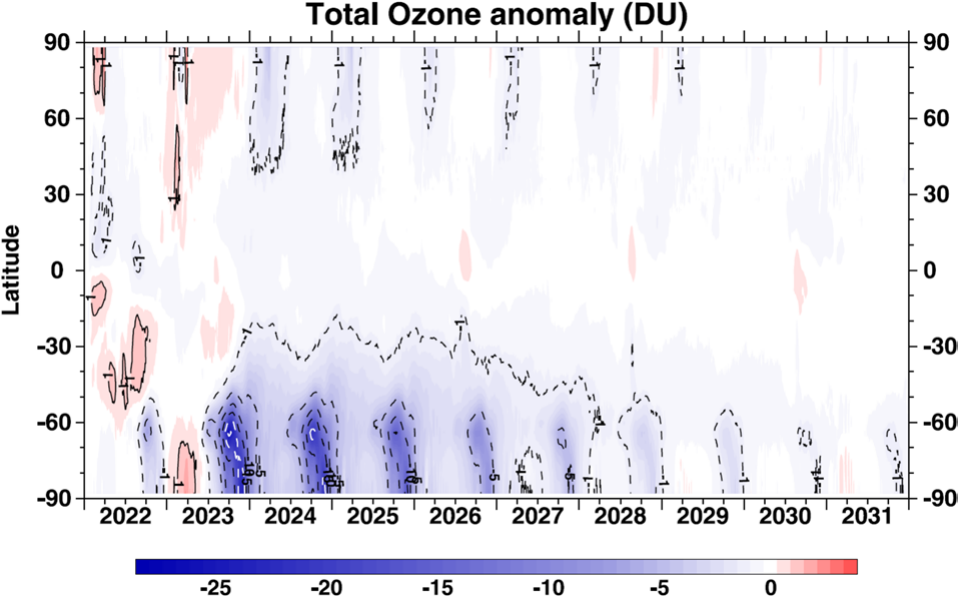
Modelled effect of the increased stratospheric water vapour concentration from the HTHH eruption on total ozone anomalies over the 2022–2031 period. Contour intervals are − 5 DU and include the ± 1 DU contours to show minor effects. Since these model calculations only quantify the effect of water vapour and not aerosols from the HTHH eruption, the full effect on total column ozone may be somewhat underestimated, at least in the first year following the eruption. Reproduced from [23] with permission from the author

On 15 January 2022, the Hunga Tonga–Hunga Ha’apai (HTHH) underwater volcano (20.5° S, 175.4° W) erupted and injected a large amount of water vapour into the stratosphere, thereby increasing stratospheric water vapour concentration by about 10% [23–25]. However, the amount of stratospheric aerosols resulting from the eruption of HTHH was small compared to other volcanic eruptions of similar magnitude (e.g., El Chichón in 1982 and Mt. Pinatubo in 1991). The atmospheric effects of the eruption, including the effects on the ozone layer, were therefore mostly induced by the excess water vapour with additional contributions from aerosols plus a small but important injection of chlorine into the stratosphere by the volcanic plume [26, 27]. Model calculations (Fig. 2) indicate that the long-term effect of this added water vapour on the ozone column is minor (i.e., less than 2 Dobson Units (DU) for the tropics and the mid-latitudes of both hemispheres). Hence, increases in UV-radiation in these regions resulting from this decrease in total column ozone are expected to be smaller than 1% over the next decade.

There was almost no effect from the HTHH eruption on the 2022 Antarctic ozone hole, because the polar vortex had already formed when the HTHH water vapour reached high southern latitudes in June 2022 and presented a strong transport barrier at its edge [28]. Whilst water vapour concentrations were higher by 20–40% in the 2023 Antarctic vortex, the effect on Antarctic ozone remained minor (less than 4 DU loss in total column ozone in September and October), because the low temperatures in the vortex limited water vapour due to dehydration. Specifically, water vapour was removed through condensation in June and July 2023 before processes that would have led to ozone depletion were impacted [29–31]. This effect was not taken into consideration in the model calculations shown in Fig. 2; hence, the decrease in total column ozone at latitudes south of –60° S was smaller in 2023 than indicated in this figure. In conclusion, no important changes in UV-radiation resulting from HTHH are currently expected for the next few years over southern high latitudes.

The effect of the HTHH eruption on global climate is also small. Since water vapour is a greenhouse gas, the excess stratospheric water burden from HTHH warmed the troposphere, whilst the aerosol layer that formed by the conversion of volcanic sulphur dioxide to sulphate aerosols reduced the solar radiation reaching the Earth’s surface, therefore cooling the troposphere for several months. Studies that discuss the effect of the HTHH eruption on temperature near the Earth’s surface are so far limited. However, the few studies that are available conclude that changes in global surface air temperature were smaller than ± 0.04 °C and will further diminish over time [32–34]. These changes in temperature are much smaller than those prevented by the Montreal Protocol summarised in our previous assessment [7]. There is evidence that the effect of HTHH on tropospheric temperatures had largely dissipated by the end of 2023 because aerosols had been removed from the atmosphere by then and the water vapour anomaly had largely dispersed in the lower stratosphere [35]. However, one study predicted larger regional changes in surface air temperature, in particular over high northern latitudes, due to changes in circulation and clouds resulting from excess HTTH water vapour [36]. The spatial temperature pattern of these changes is similar to that observed from October 2023 to January 2024 [37]. These results would have to be confirmed by additional studies before they can be considered robust.

### Consequences of the upcoming decommissioning of the Aura satellite

NASA’s Aura satellite, which became operational in 2004 and is the platform for the Ozone Monitoring Instrument (OMI) and the Microwave Limb Sounder, is scheduled for decommissioning in 2025 or 2026 because of the lack of fuel, which prevents safe deorbiting [38]. By discontinuing operations, the tracking of trace gases that affect stratospheric ozone will be severely impeded. This also affects UV data products from OMI, which are of particular importance for the assessments by the UNEP Environmental Effects Assessment Panel (EEAP) concerning environmental and health effects, and for the assessment of trends in UV-radiation. No international space agency has near-term plans for launching a spaceborne instrument capable of simultaneously observing water vapour, stratospheric trace gases relevant for ozone depletion, and the effect of volcanic eruptions at the temporal and spatial coverage provided by the Microwave Limb Sounder. The community therefore has to rely on the ageing Canadian ACE-FTS (Atmospheric Chemistry Experiment – Fourier Transform Spectrometer) and SAGE III/ISS (Stratospheric Aerosol and Gas Experiment III/International Space Station) satellites that have limited global coverage.

## Potential effects of stratospheric aerosol injection

Large volcanic eruptions can inject sulphur dioxide (SO_2_) into the stratosphere, where it reacts with water vapour to form an aerosol layer of sulphuric acid (H_2_SO_4_). This layer increases reflection of incoming solar radiation leading to cooling at the Earth’s surface. Stratospheric Aerosol Injection (SAI) is designed to mimic this process by the deliberate injection of gaseous SO_2_ into the lower stratosphere.

Model simulations, using different future climate scenarios, have considered the potential effects of SAI as well as its abrupt cessation on global and regional climate [39]. Our assessment of potential consequences for the biosphere of climate intervention via SAI is drawn from models of an immediate “strong SAI” scenario, whereby atmospheric carbon dioxide (CO_2_) concentrations remain high due to inadequate mitigation of greenhouse gas emissions (GHG), requiring continuously increasing SAI to keep surface temperatures from exceeding the 1.5° C threshold defined in the Paris Climate Agreement. These models predict that, beyond decreased ground surface temperatures, SAI would have unwanted climatic effects including depletion of stratospheric ozone [40]. This could lead to cascading environmental effects, whereby regional climate changes affect surface UV-radiation, tropospheric ozone, and precipitation.

The global distribution of stratospheric ozone is projected to change due to the effects of SAI on stratospheric chemistry and aerosol-induced heating of the stratosphere, but these changes would vary greatly with latitude, elevation, aerosol concentration, and the duration of SAI [41]. Models project that immediate implementation of SAI could produce 1–2% depletion of the total column ozone globally by the 2030s ([42]; reviewed by Hyunh & McNeill [40]). This would result in increased UV-B (280–315 nm) radiation at the Earth’s surface, but the magnitude of these increases would depend on other factors such as cloud cover [7, 43]. During the Antarctic Spring, models project that total column ozone could be reduced by 58 ± 20 Dobson Units (DU) under this SAI scenario [41]. Studies of the response of polar vegetation to ozone depletion suggest that ozone depletion of 20–40 DU could lead to at most a 13% reduction in above-ground biomass (meta-analysis; [44]). Model simulations project that in the Arctic this SAI scenario may cause up to 5% (equivalent of 20 DU) ozone depletion through the 2030s. In contrast, during winter in the Northern Hemisphere at mid-latitudes (40–60° N), it is projected that the total column ozone would increase slightly by the end of the twenty-first century [41].

Stratospheric heating by SAI could cause perturbations to global circulation patterns that, like stratospheric ozone depletion, would drive changes to the hydrological cycle, potentially disrupting ecosystem processes and the cycling of carbon and nitrogen [45]. These impacts would vary with latitude. Some studies indicate that mean global precipitation would decline by 2–3% under SAI [46], which would more than offset the expected precipitation increase due to greenhouse gas-driven climate change (e.g., [47]). However, there would be substantial regional variation in the extent and direction of changes in precipitation [48–50]. In particular, a shift in the Intertropical Convergence Zone in a pattern consistent with that following some large volcanic eruptions would be expected, including a general decrease in tropical precipitation [48, 50]. A drier Mediterranean and wetter northern Europe are projected due to a poleward shift of the storm tracks over the North Atlantic Ocean (reviewed by Visioni et al. [49])). In the Southern Hemisphere, SAI is predicted to lead to an increasingly positive Southern Annular Mode (SAM) [51, 52]. These changes could result in reductions in precipitation of up to 10% during June, July, and August in Amazonia and Patagonia [48, 49]. Changes to the position of wet and dry zones, and wind patterns and their intensity, are known to affect biodiversity and ecosystem services [53, 54]. In southern Chile, where changes in precipitation would coincide with the enhanced UV-radiation mentioned above, increased drought [47] could lead to reductions in both forest growth [55] and energy generation from hydroelectric plants [56].

In addition to the projected changes in precipitation, the reductions in surface temperatures and decreased ratio of direct-to-diffuse radiation resulting from the implementation of SAI would also likely affect ecosystems [57]. However, the ecological effects of these environmental changes, against a background of a continuing increase in atmospheric CO_2_ concentrations, are highly uncertain. Some studies suggest that large decreases in carbon uptake by ecosystems in the tropics and slight increases in carbon uptake at high latitudes could occur [58–60]. Conversely, some models predict that cooling under SAI could enhance retention of the ecosystem carbon pool, largely through reducing the rate of decomposition rather than increasing photosynthesis [59, 60]. Reduced transpirational losses of water by vegetation, resulting from projected cooling by SAI and continuing high atmospheric CO_2_ concentrations, could leave more ground water available to terrestrial and aquatic ecosystems [61]. Excluding high-latitude regions, it has also been suggested that SAI would reduce the land area burned by wildfires because of decreasing temperatures and wind speed coupled with increasing relative humidity and soil moisture [62, 63]. Cooler surface temperatures could slow the rate of permafrost thaw and thereby reduce the emissions of carbon from permafrost regions [64, 65], although the cooling effect of SAI is expected to be less pronounced at high latitudes than at mid and low latitudes [51, 52, 66].

Both the implementation and the abrupt cessation of SAI would be expected to rapidly alter the climate. This could pose a significant threat to biodiversity as organisms may be unable to acclimate or adapt to these sudden environmental changes [67, 68]). Thus, the environmental and ecological effects of SAI would depend heavily on the timing, magnitude and duration of SAI forcing.

At the present time, there are large uncertainties and unknown consequences for global ecosystems of climate interventions such as SAI. Further study is needed to identify and quantify the potential unintended consequences of SAI before it is possible to assess with confidence the viability of SAI as an approach to lower the Earth’s temperature.

## UV-B radiation-induced impacts on tropospheric photo-oxidants

Solar radiation drives the tropospheric photochemical system, which forms and destroys oxidants that transform atmospheric compounds*.* Changes in incoming UV-B radiation due to changes in stratospheric ozone affect tropospheric chemistry, with consequences for the concentrations of some important pollutants and greenhouse gases, including tropospheric ozone.

### Offsetting harmful and beneficial human health effects of increased UV-B radiation

A recent study suggests that moderate increases in UV-B radiation may increase the destruction of ground-level ozone, and thus yield health benefits that offset increases in the incidence of skin cancers [69]. A numerical model of the global atmosphere was used to study the changes in tropospheric chemistry expected from hypothetical stratospheric aerosol injections (SAI). For the scenarios considered, stratospheric ozone was depleted by 5–15%, depending on latitude, and the increased transmission of UV-B radiation caused a reduction of ambient (ground-level) ozone of about 1–3 ppbv (parts per billion by volume, equal to ca 2 μg m^−3^ at sea level).

Moch and colleagues [69] estimated that globally the increase in UV-B radiation would cause an additional 950 premature deaths per year from skin cancer, and the increase in particulate matter smaller than 2.5 μm (PM2.5) would lead to an additional 6400 premature deaths from air pollution. However, the reduction of ambient ozone would avoid ca 67,000 premature deaths per year from its inhalation. Recent estimates of the global premature mortality due to ambient ozone range widely, from ca 140,000 [70] to 1,330,000 [71] per year, with the Global Burden of Disease estimate being 254,000 [72]. The benefit estimated by Moch et al. is a large fraction of these values. This is inconsistent with the relative risk estimated by Jerrett and colleagues [73] of about 1.04 (95% CI 1.01–1.07) for a 10 ppbv increase in ambient ozone. Other estimates of relative risk are even smaller; e.g., 1.02 (95% CI 1.01–1.04) [74] and 1.01 (95% CI 0.9–1.1) [75].

A re-examination of the model results of Moch et al. [64] gives a reduction in annual deaths attributable to reduction in tropospheric ozone of 2000 to 10,600, and an increase in deaths attributable to UV-induced skin cancer of 8,100 new cases (see Box 1).

**Table:** 

Box 1. Estimates of impact on mortality
*Decrease in deaths attributable to decrease in tropospheric ozone* Using current annual deaths attributable to ambient tropospheric ozone of 254,000–1,330,000 [71, 72], and the relative risk (RR) for 10 ppbv increase in ozone [73] scaled to a 2 ppbv decrease in surface ozone, gives (2/10) × (4%) × [1, 252] = a reduction in annual deaths of 2000 to 10,600
*Increase in deaths attributable to UV-induced skin cancer (SC)* This can be calculated using the formula %Δ SC = (%Δ strat. ozone) × RAF × BAF, where %Δ SC is the percent change in deaths, %Δ strat. ozone is the percent change in stratospheric ozone, RAF is the Radiation Amplification Factor and BAF is the Biological Amplification Factor. Taking RAF ~ 1.2 [76, 77], BAF ~ 0.6 [78], and %Δ O_3_ = 10% gives %Δ SC = (10%) × 1.2 × 0.6 = 7.2% increase in deaths attributable to skin cancer. Applying this to current annual mortality due to UV-induced skin cancer of 113,000 gives 8,100 new cases per year

**Fig. 3:**
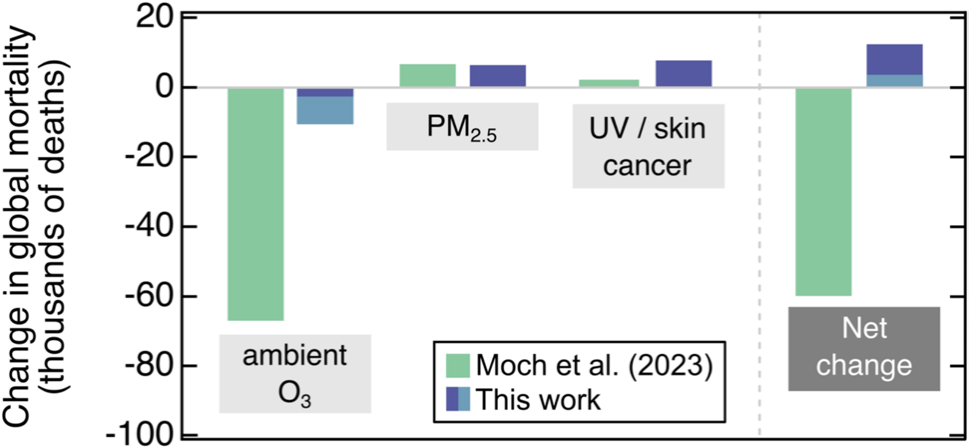
Estimated changes in global mortality as reported by Moch et al. [69] (green bars) and re-examined here (blue bars, light or dark fills showing the range of the estimates)

Figure 3 summarises this re-evaluation. The mortality attributed to a 2 ppbv increase in ambient ozone is about one order of magnitude smaller than the estimate by Moch et al., whilst the mortality change for skin cancer is larger by about a factor of 8. We did not re-evaluate the associated mortality for PM2.5, due to uncertainty about how UV-B radiation affects their formation, transformations, and removal from the atmosphere; in Fig. 3 we retain the PM2.5-related estimates given by Moch et al. [69] to compute net changes, but we emphasise that these values are highly uncertain. Our re-evaluation supports the established view that the increases in UV-B radiation lead to a detrimental net effect on human health, and is at odds with the suggestion by Moch et al. [69] that stratospheric ozone depletion may have net benefits. These inconsistencies and large uncertainties need to be resolved before drawing more meaningful comparisons between beneficial and adverse effects from anthropogenic changes in UV-B radiation.

### UV-B radiation-induced degradation of very short-lived halogenated substances affects concentrations of ozone in the troposphere

**Fig. 4:**
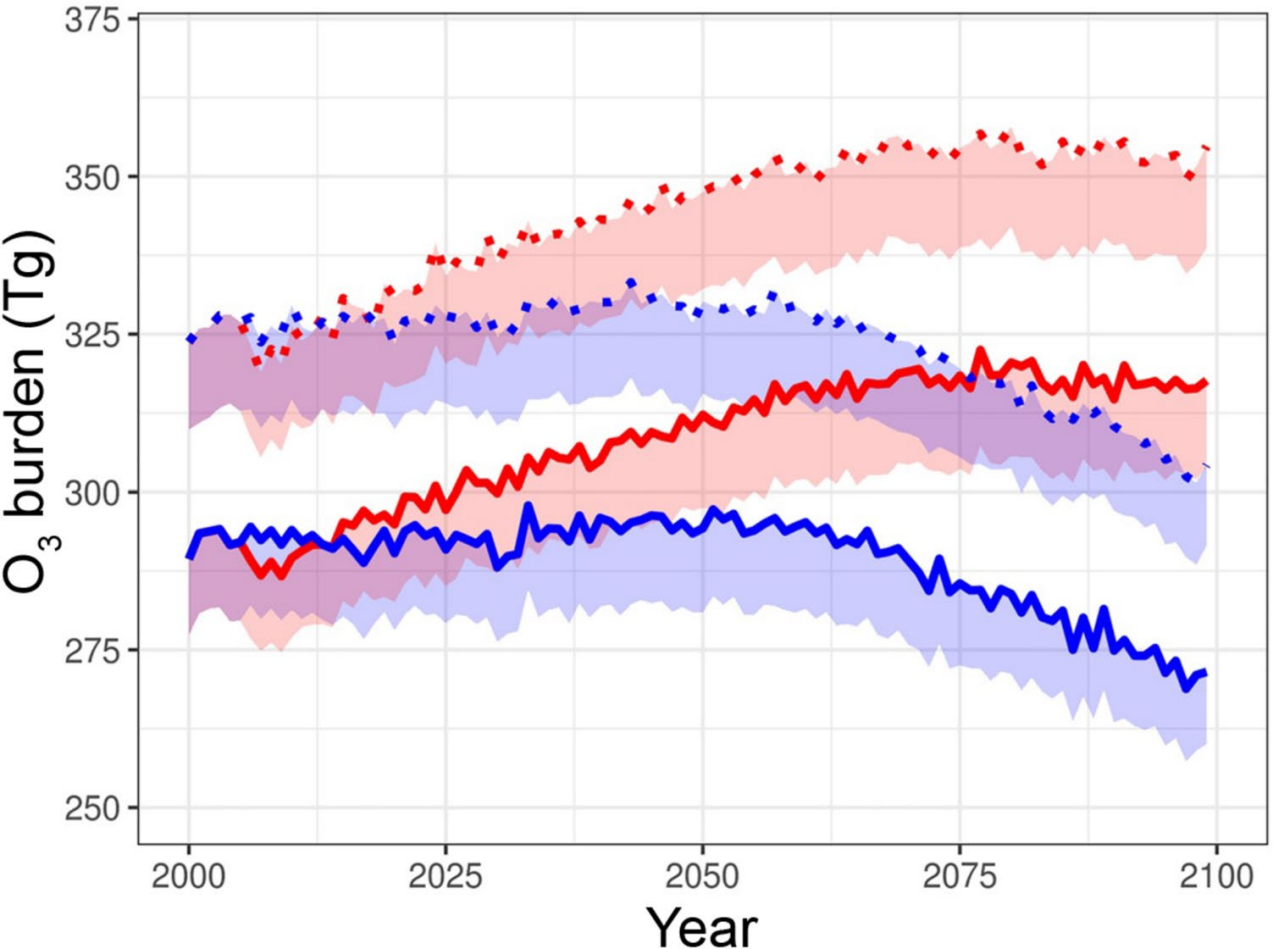
Modelled trends in tropospheric ozone *with* and *without* halogen chemistry (*solid* and *dotted* lines, respectively) for the Representative Concentration Pathways RCP6.0 and RCP8.5 (blue and red lines, respectively). Shading under traces indicate different definitions of the tropopause. RCP6.0 and RCP8.5 are representative greenhouse gas concentration pathways, which lead to radiative forcing of ca 6.0 W m^−2^ and 8.5 W m^−2^, respectively, by 2100 and global average surface temperature rises of ca 3 °C and 4 °C, respectively [82], reproduced from [80]

Very short-lived halogenated substances, including chlorinated, brominated, and iodinated VSLS (e.g., dichloromethane, CH_2_Cl_2_), are degraded via reaction with the hydroxyl radical (OH) and/or photolysis by UV-B radiation (e.g., bromoform, CHBr_3_) [79]. These processes yield reactive halogen species that can destroy ozone in the troposphere. Inclusion of halogen chemistry in models results in a considerably lower estimate of the burden of ozone in the troposphere [80]. Modelled tropospheric burden of ozone is 11–15% lower throughout the twenty-first century if natural halogenated VSLS are included (see Fig. 4) [80]. A smaller burden of ozone in the troposphere has beneficial and adverse effects. A beneficial effect is that it reduces surface warming, because tropospheric ozone is a greenhouse gas. The change in radiative forcing due to a lower tropospheric ozone burden from halogen chemistry has been simulated to be ca –0.20 W m^−2^ and –0.25 W m^−2^ for the year 2100 following the scenarios RCP6.0 and RCP8.5, respectively [81]. An adverse effect of a lower burden of tropospheric ozone is a reduced production of OH, the major tropospheric cleaning agent; this occurs, because photolysis of ozone by UV-B radiation is the principal source of OH. Lower tropospheric OH concentrations, in turn, increase the tropospheric lifetime of many gases including methane (CH_4_) and VSLS, since OH is their most important chemical sink. According to simulations [81], the increases in radiative forcing caused by increases in the CH_4_ burden due to the halogen–ozone chemistry is ca 0.1 W m^−2^ for both scenarios, RCP6.0 and RCP8.5. This study shows the importance of including tropospheric VSLS halogen chemistry in the modelling of the future radiative forcing of climate change.

## Update on the chemistry of replacements for ozone-depleting substances in relation to chemicals under the purview of the Montreal Protocol

Understanding of the atmospheric impact of replacements for ODS, including hydrofluorocarbons (HFCs), hydrofluoroethers, and halogenated olefins (HFOs), continues to improve. Trifluoromethane (HFC-23, CF_3_H) is a long-lived greenhouse gas with a 100-year global warming potential of 14,700 [5]. CF_3_H is released directly into the atmosphere, but is also produced in the atmospheric processing of a stable reaction intermediate (trifluoro-acetaldehyde, CF_3_CHO) that is formed from some ODS replacement compounds [83]. Reaction of ozone with some HFOs been shown to give CF_3_H in small yields.

Trifluoroacetic acid (TFA) is the terminal breakdown product of some replacements for ODS. TFA is persistent and ubiquitous in the environment, especially in aquatic environments. Its concentrations are increasing globally. Two other short-chain perfluorocarboxylic acids, perfluoropropanoic acid (PFPrA), and perfluorobutanoic acid (PFBA), are of relevance to the Montreal Protocol. The sources, fate, and toxicity of these acids are topics of ongoing debate.

### Formation of trifluoromethane from atmospheric degradation of replacements for ozone-depleting substances

As discussed in our previous assessments [84, 85], photolysis is an important mechanism through which CF_3_CHO is lost from the atmosphere. Photolysis proceeds by two channels, one of which results in the formation of trifluoromethane

1a $${\text{CF}}_{3} {\text{CHO}}\, + \,h\nu \to {\text{CF}}_{3} \, + \,{\text{HCO}}$$

1b $${\text{CF}}_{{3}} {\text{CHO}}\, + \,{\text{h}}\nu \to {\text{CF}}_{{3}} {\text{H}}\, + \,{\text{CO}}{.}$$

The data evaluation panel of the International Union of Pure and Applied Chemistry (IUPAC) provides evaluated quantum yields for formation of CF_3_H of Φ_1b_ < 0.003 at a wavelength of 308 nm and Φ_1b_ = 0.16 at a wavelength of 254 nm [86]. Hence, for UV wavelengths and pressures relevant for the troposphere, channel (1b) is of little importance, but for UV wavelengths and pressures in the stratosphere, channel (1b) is significant. Therefore, formation of CF_3_H in the photolysis of CF_3_CHO is expected to be important only above altitudes of 30–40 km [87]. CF_3_CHO can be formed as an atmospheric degradation product of very short-lived precursors such as HFO-1234ze, (CF_3_CH=CHF) and long-lived precursors such as HFC-143a (CF_3_CH_3_). Very short-lived precursors such as HFOs will not survive long enough in the troposphere to be transported to the stratosphere in significant amounts, and hence, the formation of CF_3_H from photolysis of CF_3_CHO is of negligible importance. Long-lived precursors such as HFC-143a (CF_3_CH_3_) are transported to the stratosphere in significant amounts; hence, the formation of CF_3_H from photolysis of CF_3_CHO could add a significant secondary contribution to the radiative forcing of these precursors. A similar conclusion has been presented in a recent report by the UNEP Scientific Assessment Panel [83].

Since our last assessment, Pérez-Peña and colleagues [88] reported the results of a modelling study and concluded that the photolysis of CF_3_CHO could be responsible for around 4–15% of the current atmospheric growth rate of CF_3_H (assuming Φ_1b_ = 0.003–0.010). However, their model erroneously used the *upper* limit for the *molar* yield of CF_3_H, reported by Sulbæk Andersen et al. [87], as the *lower* limit for the *quantum* yield (Φ_1b_ < 0.003). The *upper* limit of Φ_1b_ = 0.01 used in the model was extrapolated from a non-peer-reviewed study conducted at low-pressure, as a “realistic upper bound”. The quantum yield range of Φ_1b_ = 0.003–0.010 assumed by Pérez-Peña and colleagues [88] is inconsistent with the upper limits of Φ_1b_ < 0.0034 measured by Chiappero et al. [89] and Φ_1b_ < 5 × 10^–4^ which can be derived from the work of Sulbæk Andersen and Nielsen [90]. Thus, the model substantially over-estimated the yield of CF_3_H from the atmospheric photolysis of CF_3_CHO.

Salierno [91] suggested that trifluoromethyl radicals (CF_3_), formed during the atmospheric degradation of HFOs, could react with hydrogen-containing species such as water and methane to produce CF_3_H. This assumption is incorrect; CF_3_ radicals do not react to give CF_3_H in the atmosphere. Instead, CF_3_ radicals react with molecular oxygen to give CF_3_O_2_ radicals, in a reaction which is many orders of magnitude faster than the reaction of CF_3_ radicals with hydrogen-containing species. Salierno [91] also assumed that decomposition of CF_3_O_2_ to regenerate CF_3_ radicals is significant. This is also incorrect; the atmospheric fate of CF_3_O_2_ radicals are reactions with NO, NO_2_, HO_2_, and RO_2_ radicals [92].

There is another possible pathway for generation of CF_3_H. The reaction of tropospheric ozone with aliphatic alkenes involves a “hot channel” in which the acetaldehyde oxide Criegee intermediate can form 3-methyldioxirane. The dioxirane subsequently isomerises to acetic acid carrying enough internal energy to decompose to methane and CO_2_. An analogous process leading to CF_3_H formation has been identified by McGillen et al. [93] for the 2,2,2-trifluoro-acetaldehyde oxide Criegee intermediate, which is produced from ozonolysis of CF_3_ substituted olefins, such as HFO-1243zf (CF_3_CH=CH_2_), HFO-1336mzz (CF_3_CH=CHCF_3_), and HFO-1234ze. The implication is that the total global warming potential (GWP), established for certain halogen-substituted olefins, would be larger when including the potential for CF_3_H formation through reaction with tropospheric ozone. McGillen et al. [93] reported molar yields of 0.37–3.11% CF_3_H. The secondary contribution of CF_3_H to the radiative impact of the parent HFO is only relevant for certain halogenated olefins: e.g., for HFO-1234ze, the total GWP (integrated over 100 years) increased from 1 to 13 when including the secondary impact of CF_3_H, whilst for HFO-1336mzz (CF_3_CH=CHCF_3_), there was no effect. The inclusion of CF_3_H generated by ozonolysis does not alter our previous assessments [84, 85] that HFOs have minimal contributions to the radiative forcing of climate change.

### New measurements of concentrations of trifluoroacetic acid, perfluoropropanoic acid, and perfluorobutanoic acid in the environment

There have been several new peer-reviewed papers detailing concentrations of trifluoroacetic acid (TFA), perfluoropropanoic acid (PFPrA), and perfluorobutanoic acid (PFBA) in environmental compartments globally since the last update. In addition to the scientific literature, there has been a substantial increase in the availability of government reports that include data for these species in drinking water because of recently launched monitoring programmes in Europe (see Sect. 4.4). New data on TFA in water are summarised in the Supplementary Information (Table SI 1), along with PFPrA and PFBA when co-measured to provide context about relative amounts. TFA relative to PFPrA and PFBA is typically detected more frequently, and at greater concentrations. The current concentrations of TFA in drinking water and surface waters remain below current thresholds of concern for human and ecological health [85, 94]. Therefore, there is no change to the previous assessment of *de minimis* risk from TFA to the environment and human health [84, 85].

Recent measurements of TFA and PFBA in soil in China have been reported. Zhao and colleagues [95] sampled soils near oil refineries (*n* = 21) and from reference sites (*n* = 5). Most sites had just the surface layer collected (0–20 cm), whilst five had soil-samples taken up to 100 cm in depth. TFA (detection rate 100%) was found at concentrations of 0.11−16.4 μg kg^−1^ dry weight (dw) (median = 1.27 ng g^−1^ dw). PFBA (detection rate 100%) was found at concentrations of 0.23–4.31 μg kg^−1^ dw (median 0.96 μg kg^−1^ dw).

A comprehensive study of TFA in groundwater of various ages in Denmark found a strong relationship between the age of the water and the TFA concentration [77]. TFA was not detected in tritium-free groundwater recharged prior to 1960, indicating no natural terrestrial production or atmospheric deposition prior to this time. This is in agreement with previous ice core and freshwater measurements [96].

The German UBA (Umweltbundesamt) published a report with new measurements of TFA in samples from the Atlantic Ocean collected from surface waters (*n* = 33) and from seven distinct depth profiles (*n* = 41) in 2022–23 [97]. Concentrations in samples of surface water on a north–south transect of the Atlantic (ca 12,000 km) ranged in concentration from 260 to 306 ng L^−1^. Samples from depth profiles (up to 4590 m) had concentrations ranging from 237 to 294 ng L^−1^ with a slight decreasing trend in concentrations of TFA with increasing depth in six of the seven profiles. These results can be compared with measurements of TFA levels of 190–210 ng L^−1^ in a depth profile to 4150 m in the mid-Atlantic in 1998 [98, 99] and 28–200 ng L^−1^ in depth profiles to 5300 m in 1998 and 2002 in the North and South Atlantic [98, 99]. The origin of the substantial scatter in TFA levels reported in the depth profiles in 1998 and 2002 in the North and South Atlantic [98, 99] is unclear. The limited number of studies, different sampling sites, and data scatter preclude an assessment of trends of TFA levels in ocean water.

The mass of TFA measured in the oceans in the late 1990s and early 2000s, assuming even distribution of 200 ng L^−1^, was of the order of 500–1000 times higher than the estimated total anthropogenic TFA input to the environment (including Montreal Protocol gases, pesticides, pharmaceuticals, and industrial uses) in the period 1930–1999, hence demonstrating the contribution of one or more natural source(s) of TFA. [100].

### No discernible contribution yet to trifluoroacetic acid in the River Rhine from degradation of replacements for ozone-depleting substances

**Fig. 5:**
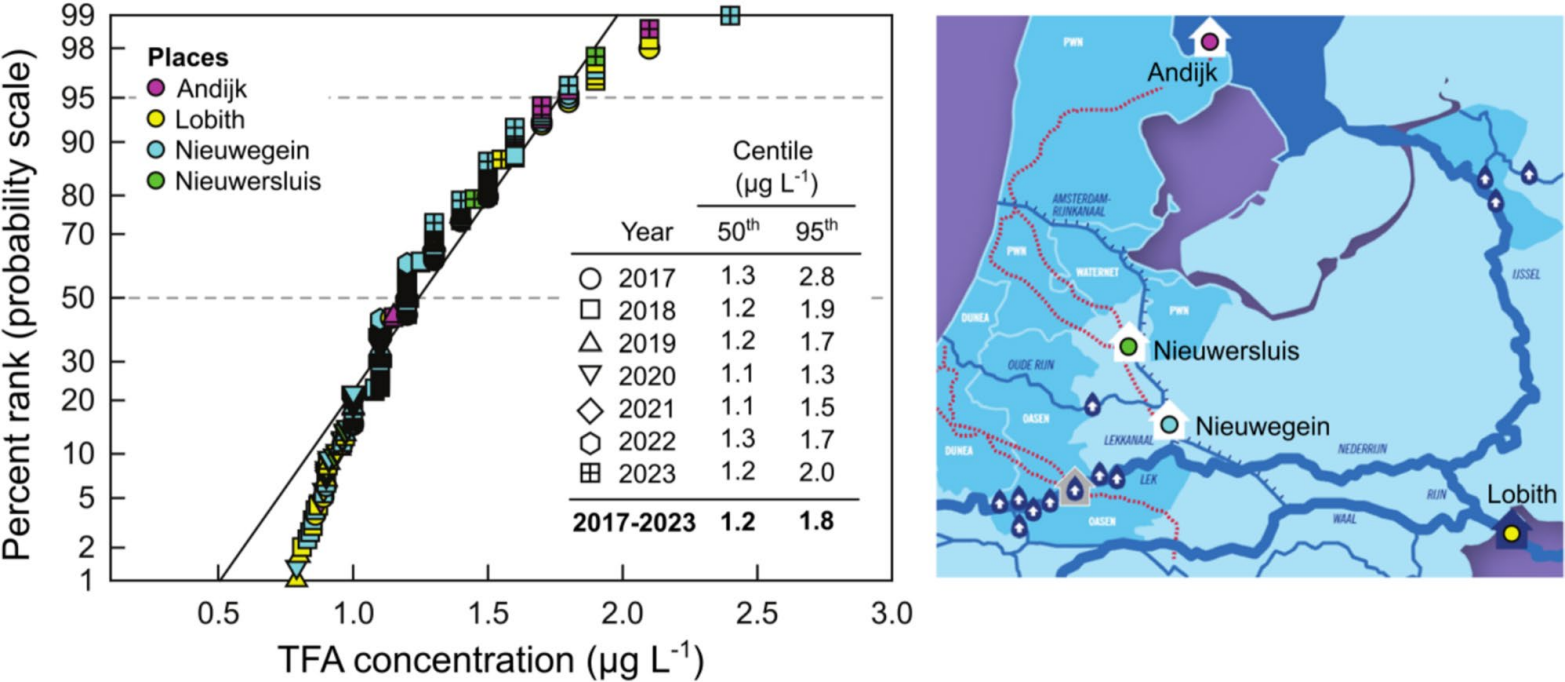
A cumulative frequency distribution of combined data from all measurements of TFA in the River Rhine (2017–2022). Data from the RIWA reports for 2017 to 2023 (*n* = 297) [101–107]

HFC-134a and HFO-1234yf are the ODS substitutes that have the largest predicted contribution to global concentrations of TFA. HFC-134a, which has been used since the 1990s as a refrigerant, aerosol propellant, and blowing agent in foams, has an atmospheric lifetime of ca 14 years. It degrades to give TFA in a molar yield of 7–20%, and its TFA-degradation product is deposited globally. HFO-1234yf was introduced as a replacement for HFC-134a in response to regulations in Europe starting in 2017. It has an atmospheric lifetime of ca 11 days, degrades to give TFA in a molar yield of 100%, and its TFA-degradation product is deposited regionally rather than globally [84]. The global average deposition fluxes of TFA from degradation of HFC-134a and HFO-1234yf in 2020 have been estimated to be 20–60 and 60 g TFA km^−2^ year^−1^, respectively [84]. The Netherlands has a monitoring programme for substances in the water of the River Rhine that conducts monthly analyses for many compounds including TFA in the river [101–107]. In Fig. 5, data from 2017–2023 for four locations (Lobith, Andijk, Nieuwersluis, and Nieuwegein) were ranked and plotted as cumulative frequency distributions for illustrative purposes. The 50th centile was calculated with the Excel < percentile.exe > function as 1.2 µg L^−1^.

Multiplying the average concentration of 1.25 μg L^−1^ measured at Lobith for 2023 with the Rhine flow rate (2360 m^3^ s^−1^) [107] gives a discharge of ca 93 tonnes of TFA in 2023. The Rhine drains a basin of around 185,000 km^2^, implying an average flux into the basin of ca 0.50 kg TFA km^−2^ year^−1^.

**Fig. 6:**
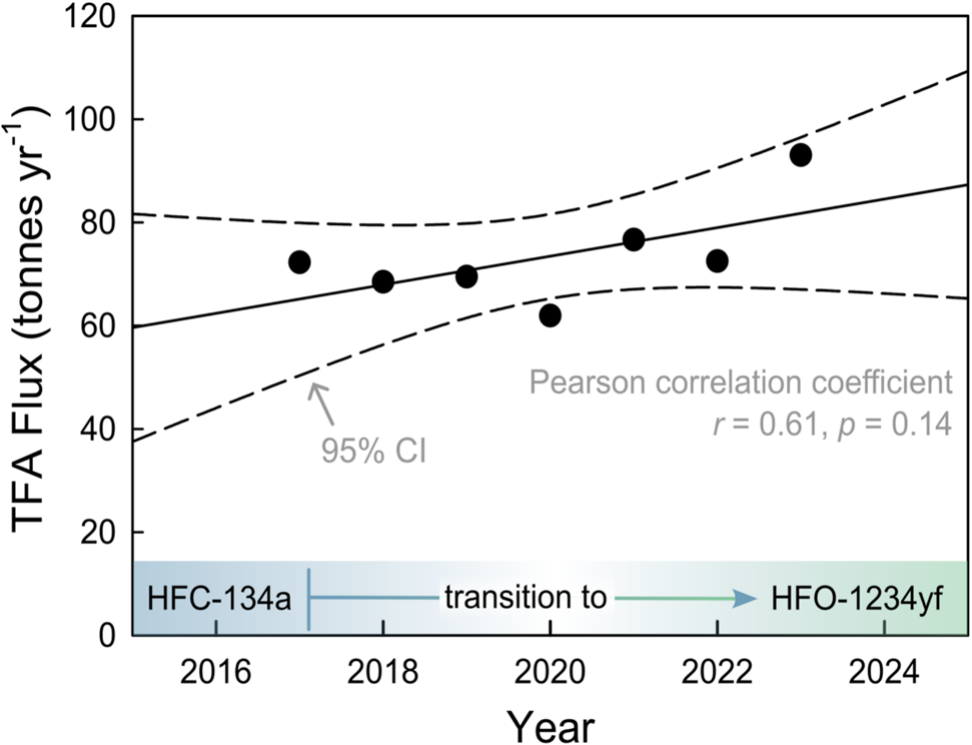
Flux of TFA in the river Rhine estimated from reported concentrations and flow rate at Lobith using data from RIWA reports for 2017–2023 [101–107]. The line through the data is a linear least-squares fit which has a slope of 2.8 ± 3.2 tonnes year^−1^

Two lines of evidence suggest that degradation of chemicals under the purview of the Montreal Protocol was not the dominant contributor to the TFA flux in the Rhine basin for 2017–2022. First, the flux from HFC-134a degradation (20–60 g km^−2^ year^−1^) is approximately an order of magnitude too small to account for the measured concentrations of TFA (ca 500 g km^−2^ year^−1^) [84]. Second, if degradation of HFO-1234yf were significant, there would have been a large increase in measured flux of TFA after HFO-1234yf was introduced to meet new vehicle regulations in 2017. However, as seen in Fig. 6, there was no statistically significant increase in measured TFA flux in the Rhine over the period 2017–2023 [106]. The absence of a temporal trend in the flux of TFA in Fig. 6 suggests either that HFO-1234yf degradation was not a major contributor to TFA in the Rhine basin for 2017–2023, or that its increasing contribution was masked by a compensating decrease in the contribution of other sources. The simplest explanation is that the observed TFA levels mainly reflect other sources, such as degradation of agrochemicals and pharmaceuticals, and/or direct emissions from industrial facilities.

Two modelling studies of the contribution of HFO-1234yf to TFA in the Rhine basin have been reported. Henne et al. [108] constructed high and low emission scenarios and found that emissions of HFO-1234yf would lead to deposition of 0.37–0.76 kg TFA km^−2^ year^−1^ in Europe in 2020. This range agrees with the flux estimated here from the observed level of TFA in the Rhine in 2023, but it is inconsistent with the absence of a trend from 2017 when HFO-1234yf was introduced into use. A report from Ramboll [109] argues that Henne et al. over-estimated emissions of HFO-1234yf and that by 2030 atmospheric degradation of HFC-1234yf would contribute 0.57 µg L^−1^ to the concentrations of TFA in the Rhine delta. This can be compared to the average of 1.25 µg L^−1^ measured in Lobith in 2023 [107]. From the available data, we conclude that there is no evidence that degradation of chemicals under the purview of the Montreal Protocol has made a major contribution to concentrations of TFA measured in the Rhine basin at this time.

### Effects of trifluoroacetic acid on ecosystems and human health

No new data on the toxicity of TFA to organisms in the environment have been reported in the literature since the last update report [84]. Effects in marine organisms and chronic exposures in freshwater organisms remain the greatest uncertainty, as noted previously [84, 110]. As reported in Table SI 1, concentrations of TFA pose a *de minimis* risk in aquatic ecosystems.

From a human health perspective, the effects of TFA salts on mammals have been reviewed previously [94, 111, 112] and these reviews have included characterisation of the risks of TFA to humans. There are no studies on the effects of dietary exposure of humans to TFA salts. However, there are some toxicity studies with TFA in laboratory animals that have been submitted to regulatory authorities, such as the European Chemicals Agency (ECHA) as part of the REACH^3^ programme. Some of these studies were reviewed [94]. However, the data were obtained by consulting laboratories for unidentified industries and are not published in the open literature. These studies were assessed by scientists at ECHA and their assessments are available in the dossier for TFA on the ECHA website.^4^ These toxicity studies are usually conducted using guidelines approved by the Organisation for Economic Cooperation and Development (OECD)^5^ and are acceptable by regulatory authorities in OECD member-countries. In addition, most of these studies are conducted according to Good Laboratory Practise guidelines that require detailed reporting of the conduct and results of studies and certification of quality assurance and quality control.

The conclusions of multiple dose studies that used the salts of TFA are summarised in Table SI 2. These included three repeated-dose studies in rats and one mode of action study that were critically evaluated by Dekant and Dekant [94]. These studies revealed effects of TFA salts on the liver including small increases in liver mass and expression of enzymes, such as alanine aminotransferase (ALT). The review [94] noted that the liver is the most sensitive organ to exposure to TFA and that mild hypertrophy of the liver and increased expression of some enzymes in the liver suggest that TFA is a peroxisome proliferator, a mechanism of action not relevant to humans [113]. Dekant and Dekant [94] also reviewed two studies on the effects of TFA on foetal development in female rats (see Table SI 2). A later study in rabbits was not available before the Dekant and Dekant paper was published. This later study is summarised in the Supplemental Information (SI) for reference, but it cannot be used in risk assessment as it lacks a no-observed effects level (NOEL).

Where NOELs were available, these were used to estimate the margin of exposure for a child (10 kg) and an adult (60 kg) listed in Table SI 1. The margins of exposure were based on the 50th centile concentrations from a compilation of values from the literature and are shown in Table SI 2. For surface waters, it was assumed that TFA salts were not removed by treatment e.g., reverse osmosis, of the water. Based on the 50th centile of exposure concentrations (see Table SI 1), the margins of exposure for TFA are in excess of a factor of 100 that would normally be used to extrapolate non-cancer endpoints from NOELs observed in chronic exposures of laboratory animals to humans. In conclusion, the risk to humans from chronic exposures to TFA in surface waters remains *de minimis* at current concentrations of TFA.

## Fate of plastic in the environment

**Fig. 7:**
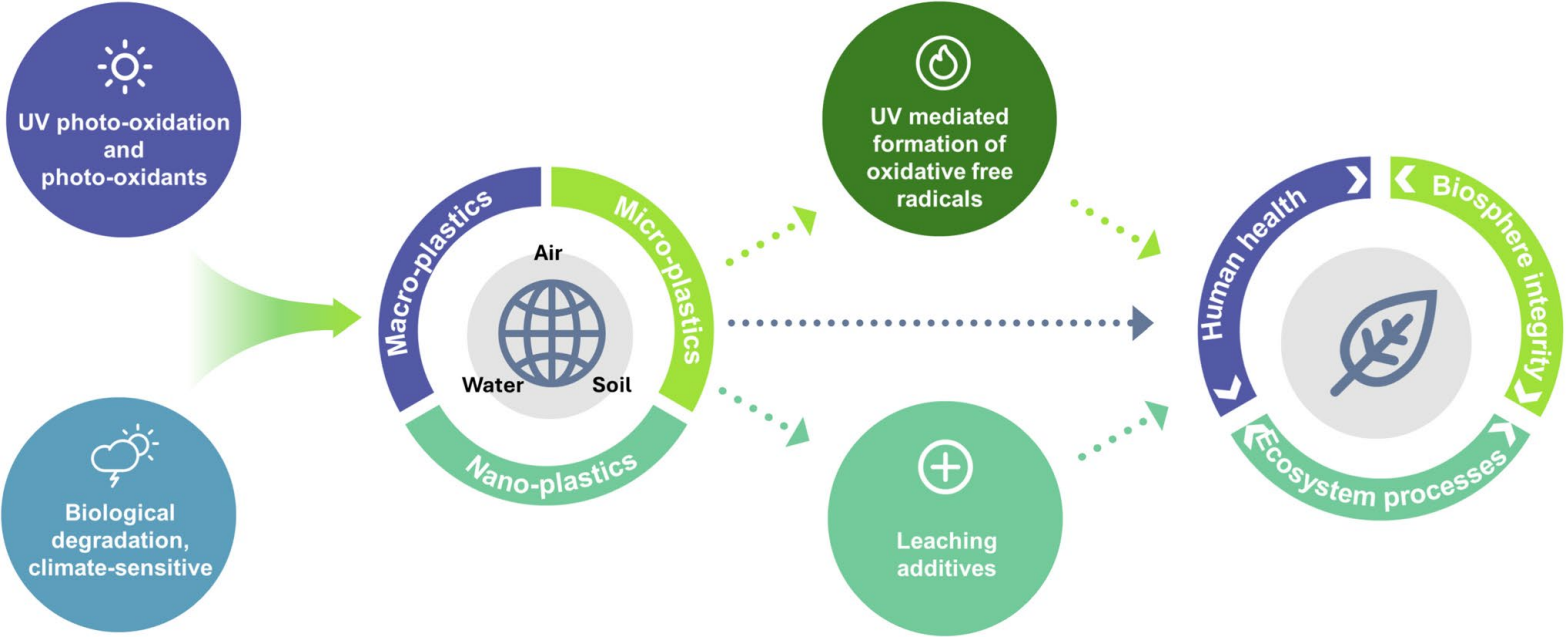
Interacting environmental processes affecting the formation of plastic particles and degradation products (such as additives) and their effects in the environment. Photo-oxidation and biological metabolism can both contribute to plastic degradation, and ultimately fragmentation. Plastic fragments and leached additives can negatively affect ecosystem processes, and organisms including humans. The finding of UV-radiation-induced persistent free radicals (i.e., reactive species present on the surface of plastics) further implicates exposure to UV-radiation with toxic effects on living organisms

In a previous assessment update for the UNEP EEAP [114], attention was drawn to formation of micro- (< 5 mm), and nano- (< 0.1 µm) plastic particles (MNPs) as a result of solar UV-radiation-driven photo-oxidative reactions (Fig. 7). MNPs originate from a range of different plastic polymers incorporating a wide variety of additives, some of which are classified as being hazardous. The ubiquitous presence of MNPs in natural environments is exemplified by their presence in, for example, arterial plaque in humans [115], semen [116, 117], and placentas [118]. Currently, limited quantitative dose–response information on UV-B radiation-driven photodegradation hinders assessments of the formation of MNP and their persistence, and hence their effects on the environment and human health. This knowledge gap also hampers research progress in the field and the implementation of the Global Plastic Treaty (https://www.unep.org/inc-plastic-pollution), which includes the impacts of MNPs.

### Airborne plastics: dispersal, exposure to UV-radiation, and climate change

Plastics are found throughout aquatic and terrestrial environments as well as in the atmosphere [114]. Location will determine the degree of exposure to solar UV-radiation, and hence the environmental fate of plastics. Over the last decade microplastics, including both fibres and fragments, have become recognised as ubiquitous pollutants in the atmosphere, where they are predicted to undergo substantial photo-oxidation due to UV-irradiation.

Since our last assessment, microplastics continue to be identified in air samples, including from remote mountainous and oceanic regions [119–121]. Several recent studies have compared the long-range atmospheric transport of microfibres and fragments [120, 122, 123]. Laboratory experiments demonstrate that microfibres have smaller settling velocities (the speed at which they fall to the ground), by as much as a factor of four, compared to spheres of comparable mass and volume, indicating that they can be efficiently transported from populated to remote regions [123]. Microplastics have been detected in clouds and rainwater [124, 125] and UV-radiation appears to increase their cloud-condensation properties [125]. However, the concentration of particles detected to date are many orders of magnitude below other cloud-forming aerosols. Typical cloud-condensation nuclei concentrations range from 1.5 × 10^8^–1.5 × 10^9^ m^−3^ at various latitudes, altitudes, and supersaturations [126]. In contrast, microplastics collected in cloud water samples were 9–10 orders of magnitude smaller (0.21 m^−3^ on average; [125]).

Significant challenges remain in quantifying the abundance of nanoplastics due to detection limits of current methods used for analysing microplastics. Mathematical modelling predicts that the number of MNPs will increase with decreasing particle diameter, following a power-law relationship in air, water and sediments [127]. However, the effects of these largely unquantified nanoplastics on atmospheric chemistry are unknown. Due to limited data on the abundance and size of particles smaller than 1 µm, which have a higher probability of penetrating to the deeper areas of lung tissues, effects on human health via respiratory inhalation are also not well established [128].

### Effects of UV-radiation on the surface chemistry of microplastics

UV irradiation causes changes to the surface chemistry of plastics which, in turn, affect transport, fate, and toxicity.

The oxidation of plastic fragments is limited to a surface layer of less than 1 µm in thickness [129]. The fragments that are exposed to UV-radiation or weathering are often more toxic to ingesting organisms than the raw plastic particles [130–132]. The increase in toxicity may arise from three processes: (i) the increase in bioavailability resulting from the reduction in particle size [131, 133, 134]; (ii) the generation of reactive functional surface groups (e.g., ketones, hydroxides, and peroxides) and persistent oxidative free radicals [135]; and (iii) the release of additives. Relatively minor surface modifications resulting from UV-radiation, such as changes in electrical potential, can modify interactions of plastics with living cells, and hence can cause toxicity [136].

### Toxicity of harmful additives from plastics

Toxicity of UV-radiation-degraded MNPs can be partially attributed to increased leaching of additives [114]. At present it is not known whether different types of plastics leach additives to the same extent. In a changing climate, leached substances can migrate, travel further in the environment, and are more likely to be bioavailable to organisms [137, 138].

Phosphorous-based flame retardants such as organophosphate di-esters (di-OPEs) and tri-esters (tri-OPEs) are added to a range of plastic consumer products. The application of OPEs is increasing since the phasing-out of brominated flame retardants [139]. OPEs are chemicals of concern that leach from degrading plastics and are found in dust, sediments, water, and air [140]. Similarly, plasticisers, such as potentially harmful phthalate esters (PAE), leach following the UV-radiation-mediated degradation of plastic [141]. PAEs readily leach when plastics are immersed in water. Unfortunately, many biodegradable plastics, deemed eco-friendly, include PAEs as plasticisers [137].

### Photo-oxidation and biodegradation

UV-radiation-induced photo-oxidation contributes to the formation of smaller plastic particles from macroplastics and removal of plastics from the environment [114]. Consistent with this, new research indicates that plastics exposed to natural solar radiation in coastal surface waters release more dissolved organic carbon compared with lower light conditions below the water surface; and the leached dissolved organic carbon can be quickly taken up by microbial communities [142]. However, oxidation and leaching rates can vary substantially amongst plastic types and with different additives [143]. Environmental factors can further modify oxidation rates, and caution is required when interpreting the results of laboratory studies as these can overestimate plastic degradation rates in natural environments [143]. Plastics can also be degraded via non-UV-radiation-mediated pathways (e.g., [144] and references therein). These non-UV-radiation-mediated degradation processes include exposure to visible radiation, or microbial or chemical pathways and are not directly relevant in the context of the Montreal Protocol. A critical and unanswered question concerns the relative contributions of UV and non-UV-radiation-mediated microbial degradation, as well as possible interactive effects between the two plastic degradation routes. This lack of information makes it difficult to infer the global importance of degradation processes for plastic removal and carbon cycling.

### Microplastic production from mechanical plastic recycling

Plastic recycling can reduce lifecycle greenhouse gas emissions and resource depletion [145]. However, there is concern that mechanical grinding of plastic waste results in point-source emission of MNPs into the environment [146]. Whilst it is not known to what extent recycling contributes to worldwide production and release of MNPs, in laboratory studies where plastics were pre-exposed to UV-radiation before mechanical grinding, production of MNPs increased by 120–180% [147]. Such unintentional particle release compromises the goal of increased sustainability via recycling, and is particularly of concern for local environments affected by point-source emissions.

## Release of additives from the ageing of materials due to solar UV-radiation and climate change: unintended consequences and counteracting technologies

**Fig. 8:**
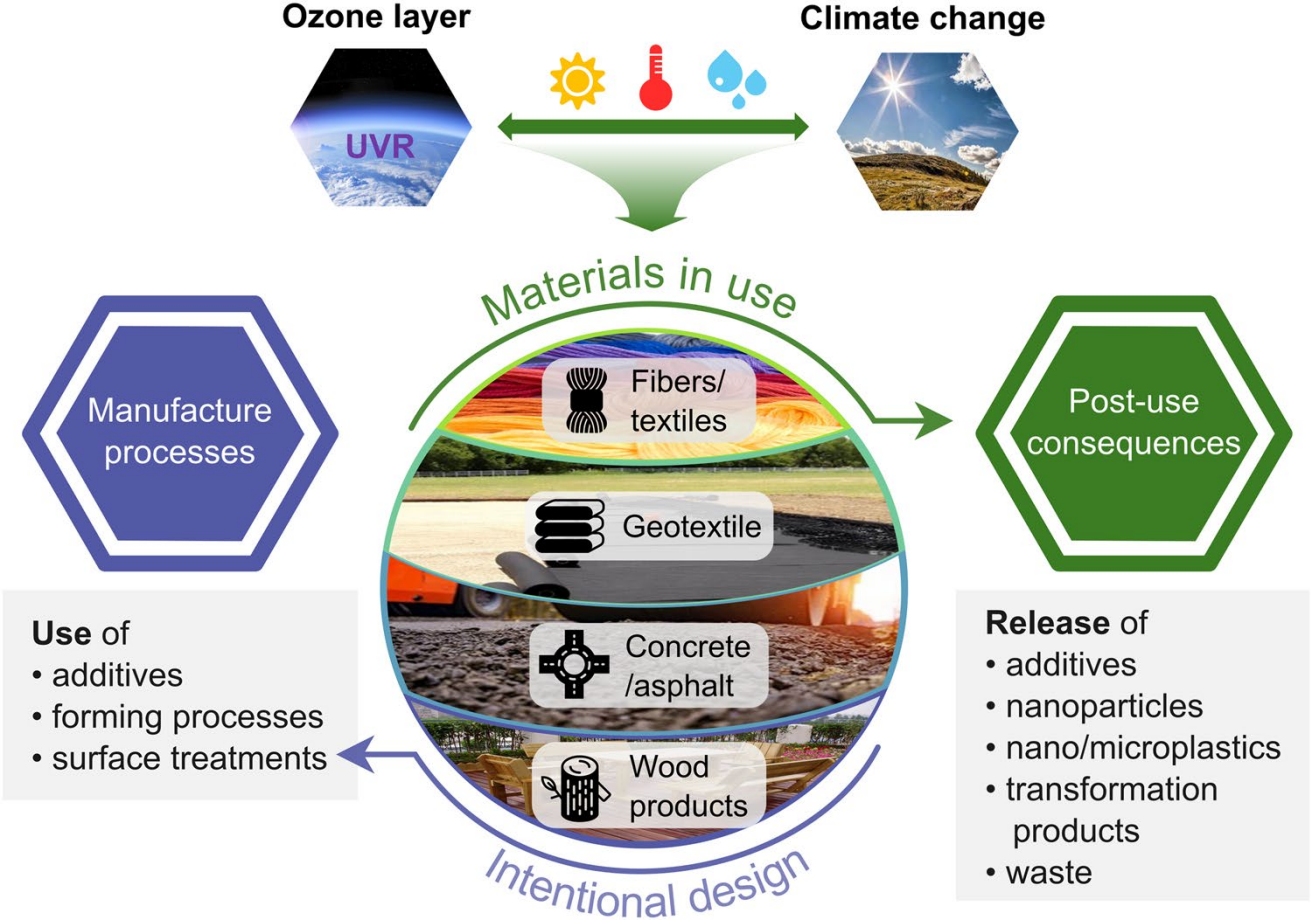
Conceptual diagram illustrating the cycle between the design, production, use, and disposal of materials. Products are intentionally designed for a specific need. To impart specific properties to the materials, manufacture processes involve, for instance, use of additives. Materials in outdoor use are exposed to solar UV-radiation and other weathering agents that cause ageing. Deterioration following ageing leads to post-use consequences that should be addressed by modifying the manufacture processes

Natural and synthetic polymeric materials used outdoors age due to exposure to solar UV-radiation and other environmental stressors, causing loss of useful service life. Changes in climate and levels of solar UV-radiation affect the rates of weathering of outdoor materials. Materials are intentionally designed to optimise their performance for specific applications, but their design considerations rarely include the post-use environmental consequences (Fig. 8).

The plastic formulations usually include additives to ensure performance, such as UV-stability, mechanical properties, and flame and heat resistance. Currently there are over 10,000 potential additives for polymeric materials [148]. It is often assumed that additives are trapped within the plastic matrix for the life of the material and the assembly is therefore benign. However, degradation of materials and subsequent release of components, including additives and micro- and nanoparticles, challenges this assumption [144]. Topical examples of these unintended adverse environmental effects due to degradation of materials are presented. Specific efforts taken in striving towards more sustainable production are also highlighted.

### Greater stability and protection against UV-radiation by engineered fibres and textiles

Textile fabrics are widely used outdoors, and to protect human skin from the harmful effects of solar UV irradiation. Nanomaterials and dyes used in fibres impart photoprotection to textile fabric, but they may enter the environment when textiles are recycled or discarded. Both physical blockers of UV-radiation, such as titanium dioxide (TiO_2_), and chemical absorbers, such as benzophenones, are commonly used in UV-protective textiles. There are serious concerns about the potential for both types of additives to leach into the environment during use, recycling, or disposal of fabric. Some chemical absorbers, such as 2,4-dihydroxy benzophenones, can break down into even more toxic products during UV irradiation in the presence of TiO_2_ nanocomposites, which act as a physical UV-blocker [149]. Nano-titanium hydride (TiH_2_) was reported to be a promising and safe alternative that blocks UV-radiation and acts as an antioxidant [150]. Dyes can also enhance the UV-screening of fibres. Functionalised synthetic acid dyes increase the UV-protective effect of cotton fabric, achieving a UV-radiation protection factor (UPF) > 100 [151]. There is a paucity of information about the UV-blocking potential of natural dyes. However, a natural dye extracted from gardenia (*Gardenia jasminoides*) can enhance the UV-screening of cellulosic fibres [152]. Other naturally derived UV-absorbers, such as vanillin [153, 154], tannic acid [155], or lignin [156], are more sustainable, safer alternatives for use in UV-protective fabrics. Physical fabric structures created by different weaving techniques also contribute to the fabric’s UV-blocking capability [157].

Aramid (aromatic polyamide) fibres are often used in space, military, and firefighting applications due to their high mechanical strength and thermal resistance. UV-absorbers, such as 2,4-dihydroxy-benzophenones [158], poly(hexamethylene guanidine), and polydopamine [159], can be grafted onto the surface of aramid fibres to make them more UV-resistant whilst limiting the leaching of the absorbers. Grafting conventional UV-blocking nano-oxides on to the aramid fibres results in improved photostability of the fibres. Coating of conventional UV-blocking nano-oxides also showed improvement in the UV-stability of aramid fibres used in engineered fabrics. Examples of such nano-oxides are TiO_2_ [160], ZnO nanowires [161], graphene oxide-melamine [162], and atomic layer-deposited aluminium oxide/TiO_2_ [163]. However, these technologies need to be evaluated further before their commercial adoption by the industry.

### Enhanced release of microfibres and additives from geotextiles

Geotextiles are polymeric fabrics widely used in civil and geoengineering applications for, e.g., soil separation, coastal reclamation, and prevention of erosion. They are usually made of non-woven polypropylene, poly(ethylene terephthalate), or polyethylene. When exposed to solar UV-radiation during transport, storage, and use, geotextiles leach additives into the environment and become brittle [164], which can lead to fragmentation. Most reported data on their durability come from accelerated ageing studies [165, 166]. For example, exposing polypropylene geotextiles to UVA-340 lamps (290–420 nm) for 35 days in the laboratory halved the average molecular weight of polypropylene and thus reduced its durability. In contrast, there was little effect of exposure to high temperature and humidity [167]. After ca 42 days of exposure to UV lamps, impermeable high-density polyethylene geotextiles used as a barrier in engineered landfills lost gas permeability due to increased crystallinity, which also caused brittleness [168]. Outdoor tests on polypropylene geotextiles support these laboratory findings. The tensile strength of polypropylene geotextil dropped by ca 30% after 5 months and 24 months in natural outdoor weathering studies conducted in Malaysia [164] and Portugal [169], respectively.

Typically, polypropylene geotextiles use carbon black and hindered amine light stabilisers (HALS) as UV-stabilisers to decrease fragmentation rates and the release of additives. Laboratory studies [170] have also shown that waterborne polyurethane–TiO_2_ emulsion coatings protect the geotextiles from UV-radiation. However, these findings have not yet been confirmed by natural weathering studies. Serious environmental concerns persist regarding the release of microfibres and additives from geotextiles made brittle by exposure to solar UV-radiation [164, 171]. The Swedish Environmental Protection Agency [172] estimates that geotextiles release up to 32 tonnes of microplastics annually in Sweden alone.

### Recycled plastics incorporated into paving and asphalt materials

Both concrete and asphalt are used in pavement construction, and there is an increasing trend to use recycled plastics in paving materials to improve their performance. Incorporating recycled plastic in concrete leads to better absorption of mechanical energy, greater thermal insulation, lighter weight, and lower water absorption by the pavement [173, 174]. Recycled plastic fibre-reinforced concrete also exhibits superior performance in the mechanical properties and durability of concrete [175]. Favourable properties, together with the need for sustainable management of plastic waste, may promote wider use of these materials. However, assessments of environmental impacts of using recycled plastics in paving materials are lacking.

Recycled plastics may also be beneficial when added to asphalt. Adding polymers to hot mix asphalt may improve its properties [176–179]. Asphalt pavements age due to photodegradation of binders, and UV-radiation-initiated ageing that advances further into the asphalt than the depth of penetration of UV-radiation [180]. However, the mechanical stability of recycled plastics within asphalt that is exposed to solar radiation is difficult to determine because of the complex composition of these composite materials [181]. Exposure of recycled polypropylene copolymers to UV-radiation and heat can cause their faster degradation [182]. The surface area represented by paving materials worldwide is considerable, and mechanical eroding of their topmost layers constantly reveals new layers for exposure to solar UV-radiation. Degradation of paving materials filled with recycled plastics may therefore have wide environmental consequences in terms of release of toxic substances. Despite the lower cost and increased sustainability in using waste plastics in pavement materials, the potential environmental impacts of the practise need to be evaluated.

### Novel wood-derived UV-stabilisers as a more environmentally friendly alternative to conventional stabilisers

Sustainability considerations and green building initiatives have necessitated greener alternatives (e.g., natural oils, plant extractives, and bio-based pigments) to replace the conventional UV-stabilisers in wood coatings. Research suggests several promising approaches. For instance, the coating of wood substrates (beech and oak) with tung oil dispersed with biocarbon increases hydrophobicity and restricts discoloration under natural weathering [183]. Coating rubberwood (*Hevea brasiliensis*) surfaces with extractives derived from bark of red sanders  (*Pterocarpus santalinus*) significantly reduced photo-yellowing under accelerated weathering conditions [184]. Extracts present in the heartwood of Siris (*Albizia lebbeck*) act as UV-absorbers and impart substantial UV-stability to both untreated and heat-treated wood [185]. Similarly, the extracts from red pine (*Pinus koraiensis* Sieb. et Zucc.) wood retarded the rate of lignin degradation, and restricted colour changes [186] on exposure to solar UV-radiation. Although these results are promising in terms of property enhancement, the economic feasibility of their use must be established before commercial exploitation.

Lignin, a plant-based organic biopolymer, is the second most abundant naturally occurring polymer after cellulose. A nanocomposite film, with lignin nanoparticles in an epoxy matrix, enhanced the UV-blocking and mechanical performance of the composite [187]. Similarly, solid lignin nanoparticles incorporated into poly(vinyl alcohol) nanocomposite films produced materials with excellent tensile strength and high UV-blocking properties [188]. Lignin/TiO_2_ nanoparticles, used as nanofillers in waterborne polyurethane wood coatings, increased the tensile strength of the coating film by ca 72%. The film also exhibited excellent UV-A and UV-B blocking of 87% and 98%, respectively [189]. These research findings provide new pathways for the development of bio-based high-performance coating materials for wood and deserve further study.

## Exposure of ecosystems to climate change and extended periods of stratospheric ozone depletion

Glacial retreat from alpine and polar ecosystems, as well as earlier seasonal snow and ice melt annually, are global phenomena resulting from climate warming. They allow for the colonisation of new ecosystems on open ground. However, these newly created habitats are subject to increased exposure to UV-B radiation. This environmental effect will have been particularly acute during exceptional recent extended periods of ozone depletion at high southern latitudes.

Additionally, new research on the photodegradation of plant material, and on photodegradation of pesticides by UV-B radiation, has increased knowledge of the mechanisms governing these processes and the cycling of the breakdown products. These processes are, respectively, important for ecosystem functioning and for their potential toxicity within the environment and food chain.

### Exposure of southern high-latitude ecosystems to high UV-B radiation

During 2020–2023, the Antarctic ozone hole persisted into early summer [190], with implications for southern high-latitude ecosystems. In southernmost South America, extended periods of Antarctic ozone depletion caused transient, extremely high UV Index values, affecting over 400,000 inhabitants and Patagonian ecosystems and biota [191]. In Antarctica, the persistence of the ozone hole into early summer coincided with unprecedented sea ice losses during 2022–2024 [192, 193]. Together with early summer snow melt, these compounding factors exposed Antarctic and Southern Ocean biota to increased UV irradiation, with potentially negative consequences for marine and terrestrial ecosystems [190]; for example, species at the base of the food chain may be particularly affected due to the increased cost of producing UV-protective compounds [190].

### Exposure of colonising species on newly uncovered ground to high UV-radiation environments

Loss of glaciers due to anthropogenic climate change will expose the ground, facilitating colonisation by pioneer species, as well as exposing new alpine and Antarctic environments to high UV-radiation [194]. Spatial analysis using the Global Glacier Evolution Model [195] suggests that by 2100, loss of glaciers could expose an area ranging from 149,000 to 339,000 km^2^, depending on the greenhouse gas emission scenario [194]. The bulk of these new post-glacial terrestrial ecosystems are in mountainous regions of Asia and north-west America (48–51%). Potentially, 7–10% of the newly exposed ground will be along the Andes and in Antarctica [194, 196]. These alpine and southern South American locations will additionally be exposed to elevated UV-B radiation due to ozone depletion in spring and early summer until the ozone hole recovers. Whilst plants and animals in alpine regions are well adapted to both high UV-B radiation and cold environments [197, 198], there is some evidence that such adaptation may reduce their capacity to adapt to warming environments [199].

### Photodegradation of plant material with consequences for carbon cycling across terrestrial ecosystems

The decomposition of dead plant material (plant litter) is a key biogeochemical process determining nutrient cycling in terrestrial ecosystems, affecting carbon storage, soil fertility, and greenhouse gas emissions. Photodegradation by shortwave radiation (UV-B, UV-A, and blue light) has been identified as a ubiquitous contributing driver of litter decomposition and carbon cycling over the Earth’s biomes [138, 200]. This occurs through direct breakdown of the lignin component of plant litter (photomineralisation) by the radiation [201, 202] and indirectly by facilitating subsequent microbial degradation (photofacilitation) [203–205].

**Fig. 9:**
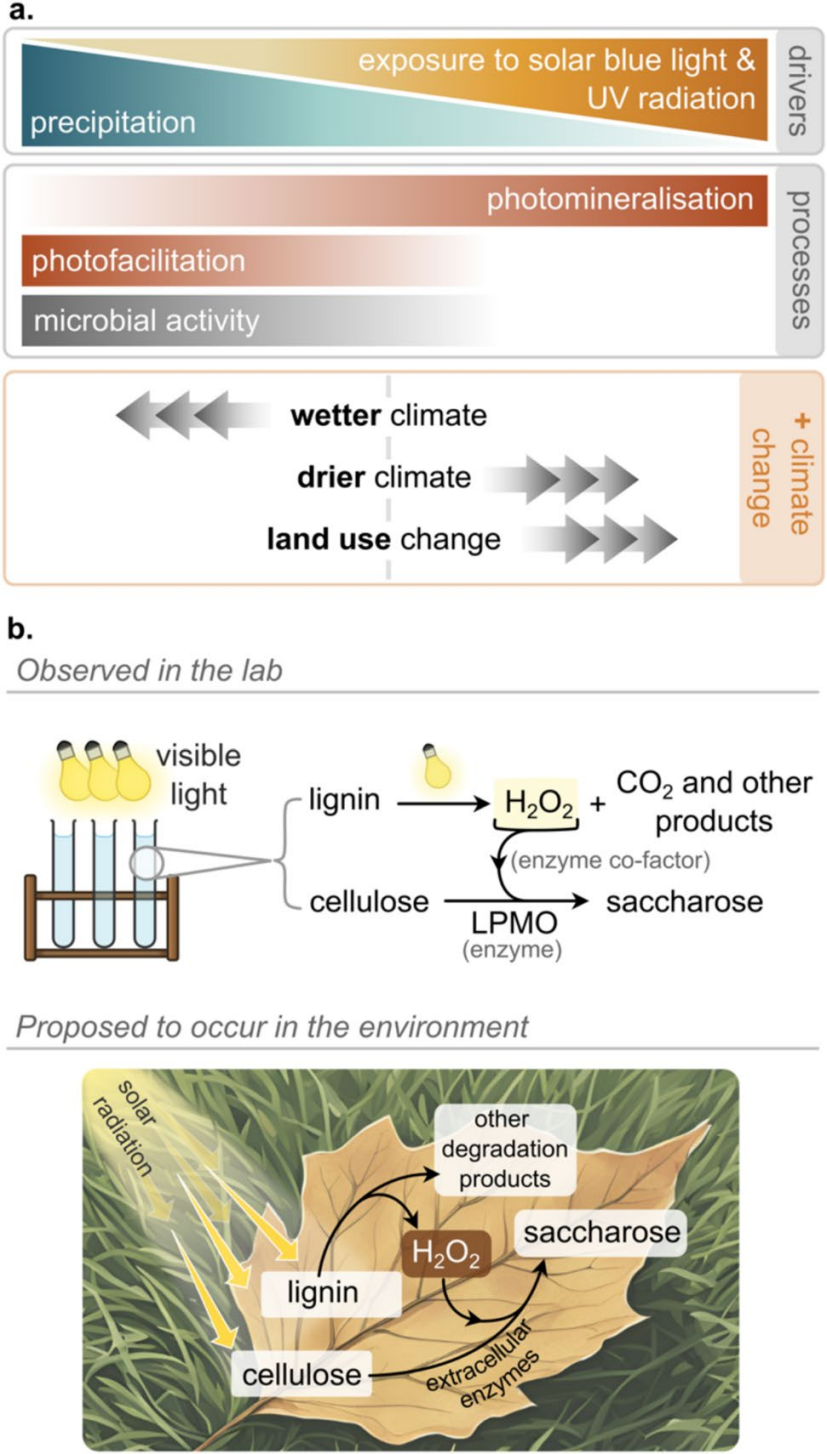
(**a**) The exposure of dead plant material (plant litter) to solar radiation and precipitation promotes photodegradation through different mechanisms. Changes in climate and land use will shift the dominance of these processes, altering the rate of litter decomposition and consequently the rate of carbon turnover in terrestrial ecosystems. (**b**) A laboratory study under controlled conditions has found that the hydrogen peroxide (H_2_O_2_) produced during photodegradation of lignin activates microbial enzymes in dead plant material (e.g. lytic polysaccharide monooxygenases—LPMO), generating carbohydrates such as saccharose. If operating in the environment, this may illustrate how the aspects of photodegradation driven by UV-B, UV-A, and blue light interact. This understanding will allow us to better assess the effects of ozone depletion on photodegradation, and thus to model the consequences for terrestrial ecosystems

Photomineralisation may be limited by the amount of photolabile compounds in litter (e.g., lignin) [205], with photofacilitation limited by water availability [203]. Consequently, climate and land-use changes modify the relative importance of photodegradation across a range of ecosystems (Fig. 9a). In general, with increasing precipitation, the primary role of solar UV-radiation shifts from photomineralisation to photofacilitation, which in turn increases leaching and microbial degradation [206, 207]. Changes in vegetation cover (i.e., deforestation and wildfires) can also shift the relative contribution of photodegradation by altering the amount of UV-radiation reaching plant litter [208–210].

A recent laboratory study on the photochemistry of photofacilitation has shown that hydrogen peroxide (H_2_O_2_), produced during lignin photo-oxidation, can enhance the activity of microbial enzymes [211]. This finding improves our understanding of how the interactions between photofacilitation and photochemical mineralisation function. If this process functions analogously in terrestrial ecosystems, it may contribute to the photofacilitation of microbial decomposition, along with enzyme-mediated degradation of lignocellulosic materials [203] (Fig. 9b). However, experiments under natural field conditions are required to verify the laboratory study. This knowledge can be applied to better assess and model scenarios of how solar radiation affects ecosystem carbon turnover and thus to predict the response of terrestrial carbon cycling to changes in stratospheric ozone, climate, and land use.

### Toxicity and persistence of pesticides and their breakdown products in the environment

The projected recovery of the stratospheric ozone layer due to the implementation of the Montreal Protocol, and contingent return of UV-B radiation at the Earth’s surface to pre-ozone depletion levels (depending on future greenhouse gas emissions), are expected to have multiple direct and indirect consequences for food supply and food quality [138]. For example, solar UV-radiation changes the persistence of pesticides in the environment [212]. This has three possible consequences: (i) decreases in UV-B radiation can reduce the photo-induced toxicity of some pesticides [213]; (ii) decreased breakdown of pesticides can reduce the decline in pesticide efficacy, preventing crop loss by extending the active lifespan of the pesticide in the field, whereby less pesticide is used reducing damage by the toxic breakdown products generated by UV-radiation; (iii) decreased pesticide breakdown leads to increased pesticide residues on, for example, food products [214]. Some of these environmental effects of UV-radiation, mediated through pesticides and their breakdown products, are counteracted by encapsulating pesticides in nano-sized carriers, such as polymers, nanoclays, and metal organic frameworks, resulting in reduced residues from photodegradation [215].

## Effects of UV-B radiation and climate change on aquatic ecosystems

Water and aquatic ecosystems provide numerous services to society, including seafood production and carbon sequestration by plankton [216]. Exposure to excess UV-B radiation can be detrimental, with some of the most significant negative effects being DNA damage and mortality [217]. However, the Montreal Protocol has protected aquatic organisms and ecosystems by preventing excessive UV-B radiation from reaching natural waters.

Climate change and alterations in stratospheric ozone modify exposure to UV-B radiation in natural waters, affecting ice cover, the depth penetration of UV-B radiation, the mixing depth of plankton, and the temperature-dependent repair of UV-induced DNA damage. All these factors were assessed in detail in the 2022 Environmental Effects Assessment Panel Quadrennial Assessment [217]. The most recent findings in aquatic systems relevant to the Montreal Protocol are assessed below.

### Climate change in the Arctic Ocean: implications for the exposure of phytoplankton to UV-B radiation

**Fig. 10:**
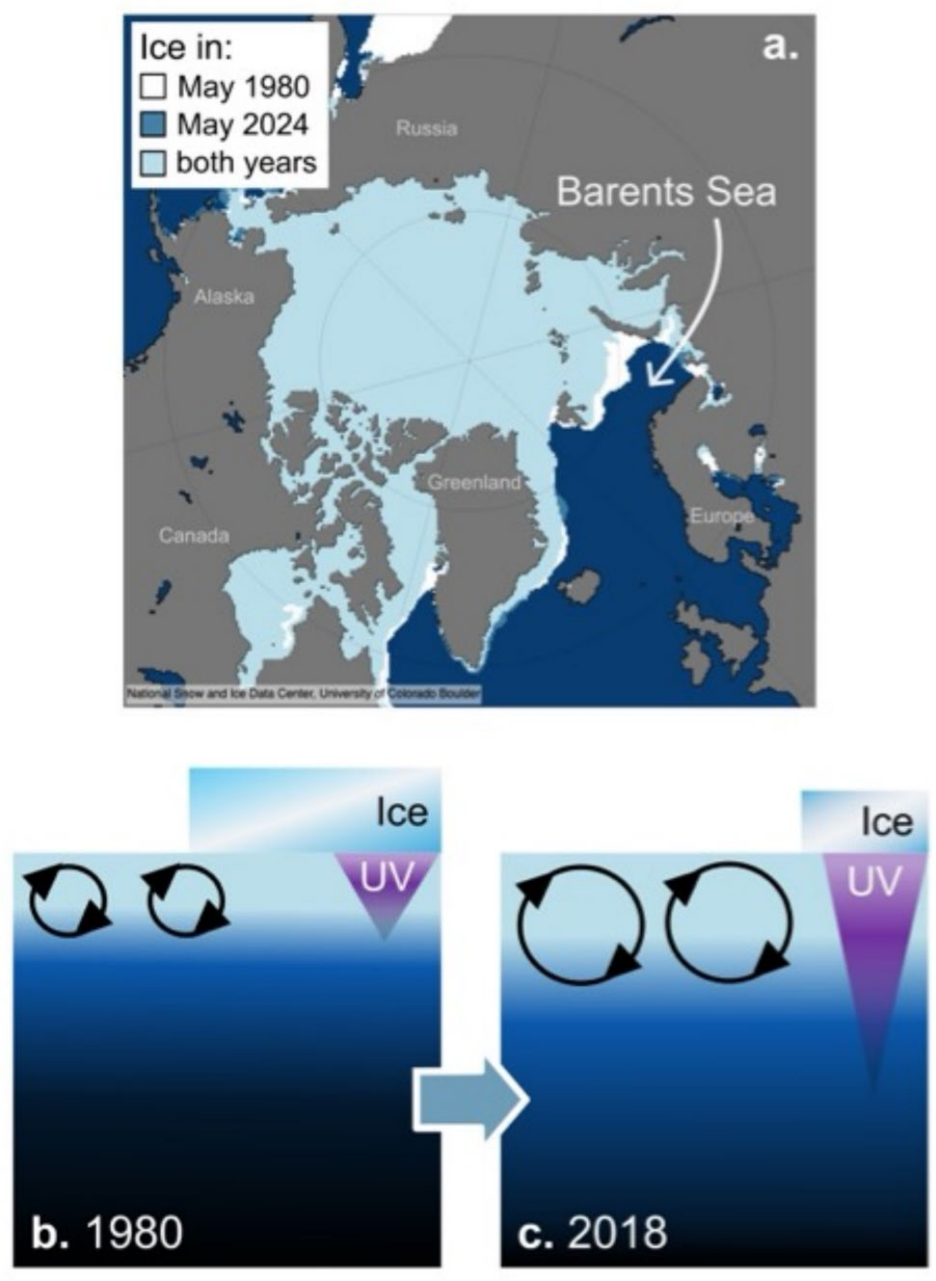
Climate change effects on the exposure of phytoplankton in the Arctic Ocean to UV-radiation. (**a**) Expansion of the late winter (May) open water area of the Arctic Ocean. White area shows extent of ice retreat between 1980 and 2024 mostly in the Barents Sea. Concurrently from 1980 (**b**) to 2018 (**c** circulation models indicate that the depth of the surface mixed layer (light blue) is increasing, reducing average exposure to UV-radiation in the mixed layer, even though the UV-radiation at the surface is increasing due to the reduction in ice cover

Carbon dioxide assimilation by phytoplankton is an important component of the global carbon cycle. Exposure to UV-B radiation can reduce this function, as well as the growth rate, and shift the biochemical composition of phytoplankton [218]. Phytoplankton circulates through the surface layer of the ocean, and since UV irradiance decreases exponentially with depth, the circulation depth determines the average exposure to UV-radiation in the surface layer (Fig. 10). In Polar Regions, surface ice cover reflects a substantial part of solar radiation before it reaches the surface water. The extent of ice cover, thickness, and depth of circulation are changing due to global warming, resulting in different exposures of phytoplankton to UV-B radiation [217]. Reductions in ice cover increase exposure to both visible (photosynthetically active radiation, 400–700 nm) and UV irradiance (including UV-B radiation). In particular, loss of ice cover will mean greater risk of exposure for Arctic phytoplankton during the typical late-winter period of stratospheric ozone depletion [18]. However, circulation models of the Arctic Ocean show a trend of progressively deeper surface mixed layers in the winter (November–May) between 1978 and 2018 [219]. The trend is the average of estimates by models participating in the latest Coupled Model Intercomparison Project, Phase 6 (CMIP6). If this trend were to continue, the deeper circulation of phytoplankton would counteract the increased exposure to UV-B irradiance due to the loss of ice cover (Fig. 10).

The deepening will be especially important in decreasing exposure in the Eurasian and Barents Sea sectors of the Arctic Ocean, much of which is already ice free in the late winter.^6^ Moreover, recovery of the late-winter stratospheric ozone depletion over the Arctic is expected during the twenty-first century (Sect. 1.4). In summary, these modelling studies suggest that deeper surface layer mixing and stratospheric ozone recovery will reduce exposure of the Arctic phytoplankton to UV-B radiation in the late winter, despite loss of ice cover (Fig. 10). However, CMIP6 models have yet to address whether changes in mixing will affect the exposure to UV-B radiation during the summer when it is expected that much of the Amerasia Basin of the Arctic Ocean will become ice free.^7^

### “Winners” and “losers” in aquatic ecosystems from the interactive effects of exposure to UV-B radiation and climate change

Climate change is, amongst other things, increasing surface temperature, CO_2_ concentration, and the frequency and intensity of climate extremes (including episodes of high rainfall as well as droughts). These factors affect how aquatic organisms and ecosystems respond to UV-B radiation. Responses occur at all trophic levels and life stages, but we first consider effects on the smallest organisms that have the least capacity to shield against UV-radiation followed by interactions affecting larger organisms [220].

#### Food web interactions at the micrometre scale

Species-specific responses determine which species are the “winners” and “losers”; i.e., whether the effects of climate change ameliorate or enhance, respectively, the negative effects of UV irradiation. For example, a population can benefit if, under climate change, it experiences less negative effects of UV irradiation than were experienced by its competitors and/or predators. In a case study of micrometre-sized prokaryotes, mostly bacteria, in a Portuguese coastal lagoon, the combination of elevated CO_2_ concentrations and exposure to UV-radiation slowed down the rate of cell division in surface incubations, whilst the population size increased [221]. This increase occurred, because the negative effect of the combined treatment was relatively less for the prokaryotes than for their consumers and/or the viruses that infect them. The same study previously reported that this combination of UV-radiation and elevated CO_2_ resulted in a shift in the dominant phytoplankton species from cyanobacteria to cryptophytes and diatoms [222]. This is another example of UV-radiation and climate change interacting to affect the composition of community species, with potential effects on aquatic food chains and carbon cycling.

#### Interactive effects at the 10–100 micrometre scale: diatoms

Other “winners” may result from ocean warming, which can enhance the defences of organisms against the effects of exposure to UV-B radiation; e.g., by increasing the rate of repair of UV-damaged DNA [223]. However, warming can also push an organism beyond its thermal limits, which can hamper defence mechanisms. At the base of marine food chains are diatoms, which are around 10–100 μm in size and are usually a main component of spring phytoplankton blooms. Several recent studies of diatoms show that warming generally results in photosynthesis becoming more resistant to inhibition by UV irradiation, although warming above the optimal temperature for growth either does not enhance resistance or, for some species, can exacerbate inhibition [224–226]. Elevated CO_2_ concentrations also have both positive and negative effects on diatoms. Limited previous work on diatoms found that elevated CO_2_ concentrations make photosynthesis more efficient, but also increases sensitivity of diatom photosynthesis to inhibition by UV-radiation (both UV-A and UV-B) [227]. These results have recently been extended to more species of diatoms [228–230], whilst information gaps remain for other phytoplankton groups.

#### Interactive effects on macroorganisms of greater than one centimetre

There is consensus that exposing both invertebrates and vertebrates to UV-B radiation has many negative effects [217, 220]. Moreover, a recent meta-analysis suggests that amphibians and fish are the most susceptible groups amongst the vertebrates [231]. However, the majority of the studies included in the meta-analysis (61 of 73) used exposure to artificial sources of UV-radiation in laboratory settings, which can be unrealistic in terms of both spectral composition and intensity. Despite this limitation, the authors conclude that vertebrates inhabiting cooler environments are particularly vulnerable to UV-B radiation due to slow rates of DNA repair. However, a recent study shows that Striped Marsh Frog larvae (*Limnodynastes peronei*), pre-acclimated to cooler temperatures, are less susceptible to damage by UV-B radiation, implying more tolerance to UV-radiation in their natural habitat than previously expected [232]. Furthermore, amphibian reproduction is dependent on ponds, and a recent study in a Brazilian system suggests that transparency to UV-radiation is low in these ponds, protecting tadpoles from exposure to UV-B radiation [233]. However, a risk with climate change is that ponds may dry out or become shallower, potentially exposing the amphibians to higher levels of UV-B radiation than they are adapted to.

Many of these recent studies of the interaction between climate change and the effects of UV irradiation use treatments that combine both UV-B and UV-A radiation. Thus, one cannot specifically ascribe how much of the responses were due to exposure to UV-B radiation and how they would change as a function of changes in stratospheric ozone. However, these studies all deal with effects known to have a UV-B component, ranging from damage to DNA, which is mostly induced by UV-B irradiation, to inhibition of photosynthesis in phytoplankton for which UV-B irradiation plays a minor role [234]. Recent findings highlight how interactions amongst organisms lead to winners or losers in response to the combined effects of UV-radiation and climate change. The net effect on a given species depends on the extent to which their predators or competitors are more or less affected.

### Susceptibility of female mosquitoes exposed to UV-B radiation to infection by Dengue

**Fig. 11:**
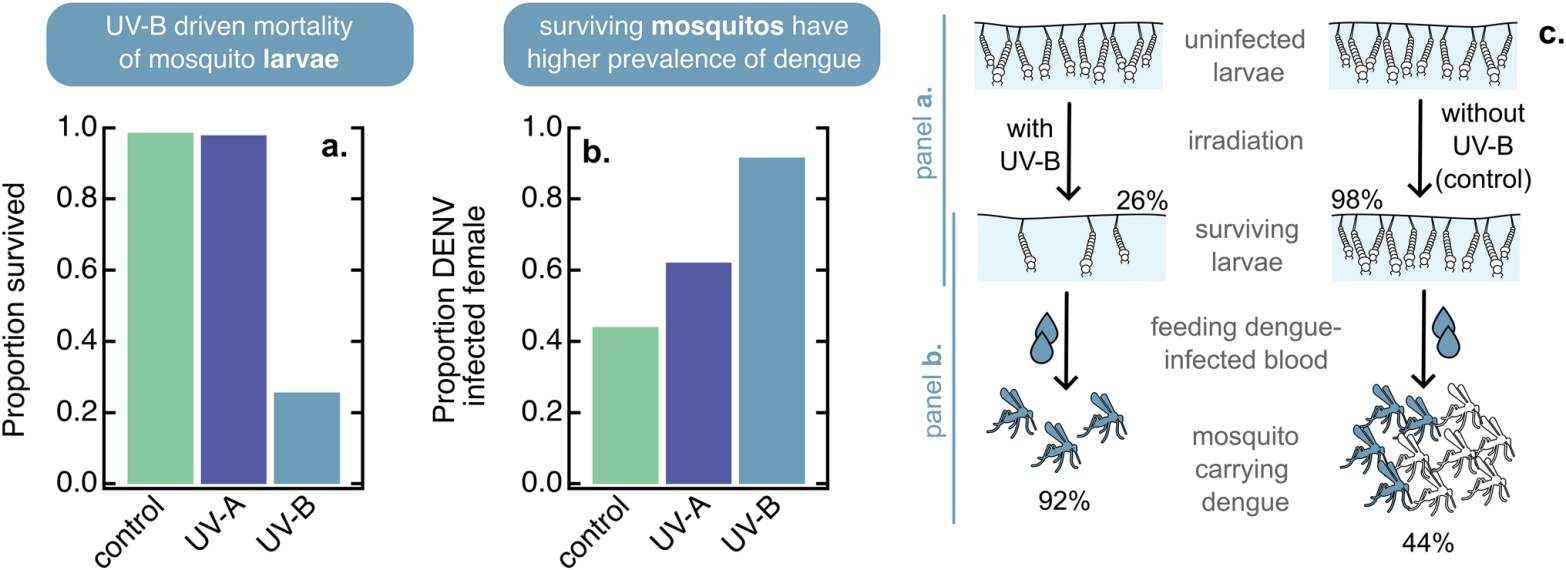
Effects of exposure to UV-B radiation on dengue infection of mosquitos. (**a**) Low survival of mosquito larvae exposed to low intensity UV-B radiation (UV Index = 1.6), (**b**) high incidence of dengue infection when surviving females are fed blood with the virus, and (**c**) these mosquitos can spread dengue infections to humans. *DENV* dengue virus

Dengue is a viral infection transmitted to humans by bites from infected *Aedes aegypti* mosquitoes, which result in an estimated 100–400 million infections and 40,000 deaths annually.^8^ Daily exposure (3 h) to UV-B + UV-A + visible radiation at low irradiance (UV Index of 1.6) from a full spectrum lamp caused high mortality of developing mosquito larvae **(**Fig. 11; [235].

The surviving females that were fed blood containing the dengue virus were much more likely to carry the dengue virus (92% infected) than females that were exposed to UV-A radiation (62%) or visible radiation only (42%) [235]. However, the viral load of dengue in the females that developed from larvae exposed to full spectrum radiation (including UV-B) was lower than for those only exposed to visible radiation. Since transmission will depend on both the prevalence of infected mosquitos and their viral load, the net effect of exposure to UV-B radiation on disease incidence needs further attention. Nevertheless, the recent research has provided additional information on how UV-B radiation might affect human health in areas where dengue virus is prevalent.

### Atmospheric factors affecting the photochemical breakdown of aquatic contaminants

Photochemical reactions control the breakdown rate of many organic pollutants in aquatic ecosystems. These reactions proceed by direct or indirect photolysis. In the former, the contaminant absorbs solar radiation and is transformed. During indirect photolysis, transformation involves reactive intermediates such as the hydroxyl (^•^OH) and carbonate (CO_3_^•−^) radicals, singlet oxygen (^1^O_2_), and excited triplet states (^3^CDOM*) that are photochemically generated by nitrate, nitrite, and chromophoric dissolved organic matter (CDOM) [217, 236].

Photochemical reactions can be modified by atmospheric conditions. For example, total column ozone (*stratospheric down to ground-level*), inorganic tropospheric pollutants, such as NO_2_, aerosols, and clouds can all affect photochemical reactions by altering solar irradiance. In the case of ozone, it mostly affects UV-B radiation that is absorbed by nitrate to yield ^•^OH and (indirectly) CO_3_^•−^, whilst the generation of ^3^CDOM* and ^1^O_2_ by CDOM is largely triggered by UV-A and visible radiation. A modelling study based on the APEX (Aquatic Photochemistry of Environmental Xenobiotics) software suggests that depletion in total column ozone from 450 to 250 DU would enhance CO_3_^•−^ and ^•^OH reactions by 10 to 25% and comparably enhance degradation of such common contaminants as carbamazepine, atrazine, diuron, acesulfame K, saturated hydrocarbons, plus phenol and anilines in organic-poor waters [237, 238]. Ozone depletion would also increase the direct photolysis of pollutants that absorb UV-B radiation (e.g., diclofenac, ibuprofen, and naproxen) [238]. In contrast, the same change in total column ozone would have little effect on ^3^CDOM* and ^1^O_2_ (+ 2–3%) generation [237] and, hence, on the degradation of contaminants, such as fenuron, isoproturon, amoxicillin, and chlorophenolates, plus phenols and anilines in organic-rich waters [238]. By comparison, NO_2_, under highly polluted conditions, and aerosols affect a much wider range of wavelengths and inhibit all indirect photoreactions [237]. As these results depend on the choice of parameters, the generality of these trends needs to be confirmed. Additionally, the modelling did not include any reduction in UV-radiation due to cloud cover, implying that photochemical reaction rates may be over-estimated. Nevertheless, measures aimed at reducing air pollution can, through consequential increased incident UV-radiation, expedite the breakdown of pollutants.

## The effects of ultraviolet radiation, including interactions with climate change, on human health

UV-radiation at the Earth’s surface has both direct and indirect effects on human health, and these can be beneficial or harmful. Exposure of individuals to UV-radiation can cause skin and eye damage (e.g., skin cancer, photodermatoses, and cataract), particularly in people with lightly pigmented skin living in sunny environments. Skin cancers include melanoma and the keratinocyte cancers [basal cell carcinoma (BCC) and squamous cell carcinoma (SCC)]; BCC and SCC are often further grouped with rare types of non-melanocytic skin malignancies and defined collectively as non-melanoma skin cancers (NMSC). UV-radiation also has benefits, such as initiation of vitamin D production and reduced risk of some autoimmune diseases. There is a vast array of indirect effects, some of which occur through interactions between UV-radiation and climate, such as changes in: (i) the risk of water- or vector-borne infectious disease (Sect. 8); (ii) food security (Sect. 7); (iii) air quality (Sect. 3); and (iv) the generation and toxicity of microplastics (Sect. 5). The Montreal Protocol has a dual role as a protector of the stratospheric ozone layer and as a climate treaty because of the regulation of ozone-depleting substances, most of which also have high global warming potential. Additionally, phase-out of some of the alternatives to the ozone-depleting substances, such as certain hydrofluorocarbons (Kigali Amendment to the Protocol) with high global warming potential, will help to further protect the Earth from increasing temperatures. The Montreal Protocol has mitigated some of the direct harmful effects of ozone depletion and potentially enabled some beneficial effects to be realised through exposure to moderate levels of UV-radiation.

### Behaviour as a driver of the trends in the incidence of skin cancer

The UNEP EEAP has monitored trends in skin cancer as one way of assessing the effects of the Montreal Protocol and its Amendments [239, 240]. However, it is difficult to disentangle the influence of changes in ambient UV-B (280–315 nm) radiation from changes in sun exposure behaviour. Melanoma and SCC have different associations with UV-radiation; SCC is strongly associated with accumulated lifetime sun exposure [241], whilst an intermittent pattern of exposure, and exposure in childhood, are more important for the development of melanoma [242, 243]. Thus, the ratio of incidence of SCC-to-melanoma can provide insights into the effects of changing patterns of exposure. A recent study examined long-term trends in the SCC-to-melanoma incidence rate ratio in seven populations with good-quality incidence data from mid-to-high latitudes, where ozone depletion and subsequent recovery have been greatest: namely, Denmark, Finland, the Netherlands, Norway, Scotland, Sweden, and Tasmania (Australia) [244]. Between 1989 and 2020, the age-standardised incidence of SCC rose faster than that of melanoma, particularly amongst women [244]. The findings are consistent with increasing cumulative exposure to UV-radiation in recent decades. The different patterns according to sex suggest that behaviour change (i.e., changes in time outdoors and/or use of sun protection; exposure to artificial UV-radiation from sunbeds), rather than a change in ambient UV-B radiation, is the more likely explanation for these trends.

Several studies have demonstrated that the incidence of both melanoma [245–247] and keratinocyte cancer [248] has decreased in younger people and in more recent birth cohorts in some high-risk populations. These declines have variously been ascribed to the success of primary prevention campaigns, a trend of young people spending more time indoors (more ‘screen’ time), or to the effects of net migration of people with more deeply pigmented skin who are less susceptible to developing skin cancer; it is likely that all three factors are contributing. A recent analysis sought to examine the contribution of changing migration patterns in Australia by examining age-specific trends in melanoma incidence from 2001 to 2021 according to ancestry. The declining incidence amongst high-risk (European-origin) Australians aged under 30 years persisted, although the change was somewhat smaller, after accounting for age-specific changes in the ancestral composition of the population over time [249]. Behaviour change in relation to sun exposure is again implicated, in this instance specifically amongst young people.

### Association between climatic factors and the incidence of melanoma

Since time outdoors underpin the amount of UV-radiation to which a person is exposed, changing climate may have an important influence on future trends in conditions such as skin cancer and cataract. A national study conducted in Canada examined the association between environmental factors and melanoma incidence, both averaged across the period 2011 to 2017, for each of ca 1000 administrative areas [250]. There was a positive association between the incidence of melanoma and average temperature, average annual number of days with heat events, mean daily vitamin D-weighted UV-radiation in summer, and green space (average normalised difference in vegetation index). For example, a one standard deviation increment in average temperature (1.5 °C) was associated with a 26% increment in the expected number of melanoma cases for a region. Conversely, higher peak annual temperature, longer duration of heat events (in days), and greater annual total precipitation were associated with lower melanoma incidence. The findings suggest that small increases in temperature are associated with increased exposure to UV-radiation (likely through more time outdoors), but larger increases have the opposite effect. The estimates need to be interpreted with caution due to the correlation between climate variables; however, these associations are consistent with an influence of temperature, light, and rain on behaviour, and provide important insights into the potential health effects of rising temperatures and other weather-related changes brought about by climate change.

### Exposure to UV-A radiation in childhood and risk of melanoma

It has long been known that sun exposure in childhood is strongly associated with risk of melanoma [242], but the relative causative contributions of UV-A and UV-B radiation are unknown. An observational study within a large United States cohort (the United States Radiologic Technologists cohort; *N* = 62,785; 80% women) linking residential exposure history and satellite-derived estimates of ambient UV-B and UV-A radiation reported that exposure to UV-A radiation during childhood was associated with melanoma after adjusting for exposure to lifetime UV-B radiation and other known risk factors [251]. In contrast, the association between childhood ambient UV-B radiation and melanoma was not apparent after adjustment for UV-A radiation. These results should be interpreted with caution due to the strong correlation between UV-B and UV-A radiation and the lack of information about sun protection (which is more likely to be promoted in an environment with high UV-B radiation). Nevertheless, the findings are consistent with the other studies that indicate that UV-A radiation may contribute to the development of melanoma [252], and highlight the need for sun protection that covers both UV-A and UV-B radiation.

### Occupational exposure and deaths from non-melanoma skin cancer

The World Health Organization and the International Labour Organization recently estimated global, regional and national burdens of non-melanoma skin cancer attributable to occupational exposure to solar UV-radiation [253]. In 2019, 1.6 billion workers were exposed to UV-radiation during their work, and 65,440 deaths from non-melanoma skin cancer occurred worldwide. Approximately 30% of both deaths (18,960) and disability-adjusted life years (0.5 million) were attributable to occupational exposure to UV-radiation.

### Balancing the harms and benefits of sun exposure

Exposing the skin to solar radiation has both harms and benefits. The balance of these is not the same for all people, but most sun protection policies (with few exceptions [254]) present a ‘one-size-fits-all’ message that does not take account of the underlying risks of skin cancer and vitamin D deficiency. A new position statement recommends providing Australians with advice tailored to their underlying risk of skin cancer [255]. It advises that everybody, other than those at high risk of skin cancer, should aim to obtain a vitamin D-effective dose of UV-radiation on most days of the week, under the assumption that this will deliver benefits in addition to vitamin D production. The position statement includes advice about the time required to obtain a vitamin D-effective dose according to geographic location, month of the year, time of day, and clothing for people with skin types I–IV (very pale to light brown sun-sensitive skin) *vs* skin types V and VI (dark brown to black sun-tolerant skin) [256]. The model underpinning these calculations uses the results of a meta-analysis to estimate the average dose of vitamin D-weighted UV-radiation needed to maintain a person’s existing 25-hydroxy vitamin D concentration [257]. Thus, the recommended times outdoors assume sufficient vitamin D status and cannot be applied to people who are vitamin D deficient.

It is important to note that more knowledge is needed about the wavelengths, dose, and patterns of exposure to UV-radiation that are associated with benefits such as reduced risk of some autoimmune diseases, or release of chemicals such as nitric oxide that may reduce the risk of cardiovascular disease. Such information will enable the balance of harms and benefits to be more precisely modelled along with the potential consequences of changes in UV-radiation.

### Chronic exposure to low-dose UV-B radiation and DNA damage

Exposing the skin to UV-B radiation is the primary cause of cyclobutane pyrimidine dimers (CPDs), a type of DNA damage that, if not accurately repaired, can lead to skin cancer. There are a number of mechanisms that skin cells use to prevent these DNA lesions being converted into harmful mutations, including nucleotide excision repair, and apoptosis (programmed cell death). There have been relatively few studies that have examined the effect of exposure to chronic low-dose UV-radiation, rather than to a single higher dose [258, 259]. A new study exposed a human keratinocyte cell line to 15 doses of 0.75 standard erythemal doses (SEDs), delivered every 12 h; this chronic low-dose regimen resulted in the accumulation of CPDs that were not repaired [260]. Further, this chronic low-dose regimen reduced removal of CPDs generated by a single high dose of UV-radiation, without increasing the rate of UV-induced cell death. Experiments in cell lines are not necessarily directly relevant to in vivo situations, and 12-hourly dosing does not represent normal human behaviour. Nevertheless, these results underscore the detrimental effects of repeated exposures to sub-erythemal (non-sunburning) doses of UV-radiation and emphasise the importance of continued protection of the ozone layer.

### Exposure to UV-radiation and ocular melanoma

The association between exposure to UV-radiation and cataract and pterygium is well established, but the link with ocular melanoma is unclear. This may be partly because small sample sizes in previous studies have precluded investigations of ocular melanoma according to the location within the eye. A new study, based in the United States National Cancer Institute’s Surveillance, Epidemiology, and End Results (SEER) programme, used county-level, satellite-based UV-radiation measures as an indicator of ocular exposure [261]. Whilst higher ambient UV-radiation was not associated with total ocular melanoma, those in the highest UV-radiation quartile were at higher risk of melanomas occurring in the iris or ciliary body (i.e., in the front of the eye, where 17% of ocular melanomas arise). These are also the sites known to be exposed to UV-radiation (posterior structures are largely shielded by the cornea and lens) and show evidence of UV-radiation signature mutations [262].

### Association between prenatal and early life exposure to solar radiation and the risk of allergies in childhood

Sun exposure is known to have an immunomodulatory effect, through induction of vitamin D and potentially other pathways, with possible benefits for allergic and some autoimmune conditions. The importance of early life exposure was exemplified by a study of 2260 children born in one of five maternity hospitals in Paris between 2003 and 2006 who underwent examinations at 18 months and/or 8 years of age [263]. There was evidence that higher ambient solar radiation during pregnancy and the first year of life was associated with reduced allergen sensitisation at 8–9 years of age [e.g., the odds ratio for sensitisation to at least one inhalant allergen per one interquartile range increase in radiation was 0.49 (95% confidence interval 0.27–0.87)]. There was a stronger association with prenatal maternal exposure than with postnatal exposure of the infant. The effects were most evident in children whose mothers had been supplemented with vitamin D during pregnancy, suggesting possible synergy between exposure to solar radiation and vitamin D. The findings highlight the possible benefits of exposure to solar radiation in early life and emphasise the need to understand the mechanisms underpinning these effects. If vitamin D is entirely responsible, supplementation is an appropriate approach to reduce the risk of allergic disease, but if there are vitamin D-independent mechanisms, sun exposure may be required.

## Supplementary Information

Below is the link to the electronic supplementary material.

Supplementary file1 (DOCX 50 KB)

## Data Availability

All data generated or analysed are either included in this published article or part of the analyses of papers cited.

## Abbreviations

ACE-FTS: Atmospheric Chemistry Experiment-Fourier Transform Spectrometer
BCC: Basal cell carcinoma
CDOM: Chromophoric dissolved organic matter
CPD: Cyclobutane pyrimidine dimers
DU: Dobson Unit
EEAP: Environmental Effects Assessment Panel
HFC: Hydrofluorocarbon
HFO: Hydrofluoroolefin
HTHH: Hunga Tonga–Hunga Ha’apai
IPCC: Intergovernmental Panel on Climate Change
MNPs: Micro- (< 5 mm), and nano- (< 0.1 µm) plastic particles
NOEL: No observed effects level
ODS: Ozone-depleting substances
OH: Hydroxyl radical
OMI: Ozone Monitoring Instrument
PFAS: Per- and polyfluoroalkyl substances
PM: Particulate matter
RCP: Representative Concentration Pathway
SAGE III/ISS: Stratospheric Aerosol and Gas Experiment III/International Space Station
SAI: Stratospheric Aerosol Injection
SAM: Southern Annular Mode
SCC: Squamous cell carcinoma
SSP: Shared Socioeconomic Pathway
TFA: Trifluoroacetic acid
UNEP: United Nations Environment Programme
UV: Ultraviolet
UV-A: Ultraviolet radiation with wavelengths 315–400 nm
UV-B: Ultraviolet radiation with wavelengths 280–315 nm
VSLS: Very short-lived substances

## Footnotes

1 Shared Socioeconomic Pathway (SSP) scenarios describe a range of plausible trends in the evolution of society over the twenty-first century and were adopted by the Intergovernmental Panel on Climate Change (IPCC) for its sixth Assessment Report. The SSPs describe five climate futures (SSP1–SSP5) that are combined with assumed amounts of greenhouse gases that are emitted in years to come. The CMIP6 simulations are based on seven SSPs (SSP1-1.9, SSP1-2.6, SSP2-4.5, SSP3-7.0, SSP4-3.4, SSP4-6.0, and SSP5-8.5), which are named after a possible range of radiative forcing values in the year 2100 relative to pre-industrial values (1.9, 2.6, 4.5, 7.0, 3.4, 6.0, and 8.5 W m^−2^, respectively), and have some equivalence to the Representative Concentration Pathways or RCPs used in IPCC’s fifth Assessment Report and our previous assessments.

2 The SAM is the leading mode of Southern Hemisphere extratropical climate variability describing a seesaw of atmospheric mass between the mid-latitude and high latitude, with corresponding impacts on the strength of the circumpolar westerly winds. A positive SAM index corresponds to a poleward shift of the maximum wind speed, which results in weaker-than-normal westerly winds in the southern mid-latitudes.

3 Registration, Evaluation, Authorisation and Restriction of Chemicals. A *European Union regulation dating from 18 December 2006.*

4 https://chem.echa.europa.eu/

5 https://www.oecd.org/en/topics/sub-issues/testing-of-chemicals/test-guidelines.html

6 https://nsidc.org/sea-ice-today/sea-ice-tools/sea-ice-spatial-comparison-tool

7 https://www.eea.europa.eu/en/analysis/indicators/arctic-and-baltic-sea-ice

8 https://www.who.int/news-room/fact-sheets/detail/dengue-and-severe-dengue
