# Supplementary material for: Environmental consequences of interacting effects of changes in stratospheric ozone, ultraviolet radiation, and climate: UNEP Environmental Effects Assessment Panel, Update 2024
Source: Photochem Photobiol Sci. 2025 Mar 17;24(3):357–92. doi: 10.1007/s43630-025-00687-x (PMC11971163; doi:10.1007/s43630-025-00687-x)
Supplement: Supplementary file 1 — Supplementary file1 (DOCX 50 KB) [file 43630_2025_687_MOESM1_ESM.docx]

# Supplementary Information for Section 4

# Update on the chemistry of replacements for ozone-depleting substances in relation to chemicals under the purview of the Montreal Protocol

Mads P. Sulbæk Andersen*^1,2^, Timothy J Wallington^3^, Mark L Hanson^4^, Barbara Sulzberger^5^, Tamara Schikowski^6^, Keith R Solomon^7^, Sasha Madronich^8,9^

^1^Department of Chemistry and Biochemistry, California State Univeristy, Northridge, CA, USA

^2^Department of Chemistry, University of Copenhagen, Copenhagen, Denmark

^3^Center for Sustainable Systems, School for Environment and Sustainability, University of Michigan, Ann Arbor, MI, USA

^4^Department of Environment and Geography, University of Manitoba, Winnipeg, MB, Canada

^5^Retired from Eawag: Swiss Federal Institute of Aquatic Science and Technology, Dübendorf, Switzerland

^6^Working Group Environmental Epidemiology, IUF-Leibniz Research Institute for Environmental Medicine, Düsseldorf, Germany

^7^School of Environmental Sciences, University of Guelph, Guelph, ON, Canada

^8^Atmospheric Chemistry Observations and Modeling, National Center for Atmospheric Research, Boulder, CO, USA

^9^USDA UV-B Monitoring and Research Program, Colorado State University, Fort Collins, CO, USA

**Corresponding author: Mads P Sulbæk Andersen, sulbaek.andersen@csun.edu*

This supplement provides more detailed information on Sect. 4 (**Update on the chemistry of replacements for ozone-depleting substances in relation to chemicals under the purview of the Montreal Protocol**) of the main text.

Since the publication of the last comprehensive assessment (*Madronich et al.,*2023) of the Environmental Effects Assessment Panel (EEAP) of the Montreal Protocol under the United Nations Environment Programme (UNEP), measurements in water samples of trifluoroacetic acid (TFA), as well as perfluoropropanoic acid (PFPrA) and pefluorobutanoic acid (PFBA) when available simultaneously, have been reported (see Table SI 1). Recent toxicity studies of TFA salts in rats, rabbits, and relevance to humans are presented in Table SI 2.

**Table SI 1.** Summary of TFA in water samples reported in the peer-reviewed literature since the 2023 Assessment update [[1](#_ENREF_1)]. Concentrations of PFPrA, and PFBA are provided when co-sampled. In addition, summary reports from water authorities from Europe are included as examples of the amount of data that will be available in the future.

| Compound | Reference and Country | | | | | | Range of Margin of Exposure for Human Health^a^ | Range of Margin of Exposure for Ecological Health^b^ |
| --- | --- | --- | --- | --- | --- | --- | --- | --- |
|  | [[2](#_ENREF_2)]:  Spain | [[3](#_ENREF_3)]: Canada | [[4](#_ENREF_4)]:United States | [[5](#_ENREF_5)]: Denmark | [[6-12](#_ENREF_6)]: Germany and Holland | [[13](#_ENREF_13)]: Norway and Sweden |  |  |
| TFA | GW: 436.8 ng L⁻¹  RW: < 50 ng L⁻¹ | RW and RWA: 4.6 to 220 ng L⁻¹ | DW: 102 to 527 ng L⁻¹  WW: 741 to 131,200 ng L⁻¹ | GW: not detected (LOD of 30 ng L⁻¹) to 1620 ng L⁻¹ | RW: 730 to 3,000 ng L⁻¹ | DW: 70 to 720 ng L⁻¹ | Adult: 100,000 to 1,363,636 | 2,100 to 28,366 |
|  |  |  |  |  |  |  | Child: 33,333 to 454,545 |  |
| PFPrA | - | - | DW: not detected (LOD of 1.5–2.6 ng L⁻¹) to < 5 ng L⁻¹ (LOQ)  WW: 11.4 to 11,084 ng L⁻¹ | - | - | - | Adult: > 3000 | - |
|  |  |  |  |  |  |  | Child: > 500 |  |
| PFBA | GW: < 50 ng L⁻¹  RW: < 50 ng L⁻¹ | RW and RWA: 0.85 to 33 ng L⁻¹ | DW: not detected (LOD of 1.5–2.6 ng L⁻¹)  WW: < 5 ng L⁻¹ (LOQ) to 52 ng L⁻¹ | - | - | - | Adult: > 600 to > 909 | - |
|  |  |  |  |  |  |  | Child: > 200 to > 303 |  |

A dash indicates not reported or not applicable. GW, ground water; RW, river water; RWA, RW near fire-training and fire-equipment testing areas of airports; DW, drinking water; WW, municipal, industrial and hospital wastewater effluent.
^a^ The threshold for effects used for TFA was the no-observed effects level (NOEL) of 10 mg kg^-1^ day^-1^ from long-term toxicity studies in rats [[14](#_ENREF_14)]; for PFPrA the US EPA non-cancer reference dose was 0.0005 mg kg^-1^ day^-1^ [[15](#_ENREF_15)], and for PFBA, 0.001 mg kg^-1^ day^-1^ [[16](#_ENREF_16)]. The consumption of water per day was 1 L and

2 L day⁻¹ for a child (10 kg) and an adult (60 kg, respectively, as per WHO assumptions. These were compared to the greatest reported concentrations in GW, RW, and/or DW.
^b^ The threshold for ecological effects used was the 5^th^ centile of the TFA species sensitivity distribution of 6.3 x 10^6^ ng TFA salt L⁻¹ (Figure 15 in [[1](#_ENREF_1)]) for greatest reported RW and RWA measurements.

Table SI 2. Summary of the conclusions of repeated dose toxicity studies on the salts of TFA and relevance of the results to risk of TFA salts to systemic and developmental toxicity in humans.

| **Test species and test guideline or protocol** | **Exposure Duration (days)** | **NOEL (mg kg bmˉ¹ day⁻¹)** | **Margin of exposure** | | **Reference** |
| --- | --- | --- | --- | --- | --- |
|  |  |  | **Child** | **Adult** |  |
| Rat extended dev. tox. study, OECD TG443 | 70 | **242**–265 | 2.02 x 10^6^ | 1.65 x 10^7^ | [[p 1071 in 14](#_ENREF_14)] |
| Rat prenatal dev. tox. study, OECD TG414 | 12 | **150** | N/A | 2.67 x 10^7^ | [[p 1072 in 14](#_ENREF_14)] |
| Rabbit dev. tox. & teratogenicity, OECD TG414 | 22 | Dam-180  Foetus < 180 | N/A | N/A | [[17](#_ENREF_17)] |
| Rat repeated dose study, OECD TG407 | 28 | Males **1,315** mg kg bw⁻¹ day⁻¹  Females 1,344 mg kg bw⁻¹ day⁻¹ | 1.10 x 10^7^ | 3.04 x 10^8^ | [[p 1070 in 14](#_ENREF_14)] |
| Rat 90-day study,  OECD TG408 | 90 | Males **10** mg kg bw⁻¹ day⁻¹  Females 12 mg kg bw⁻¹ day⁻¹ | 8.33 x 10^4^ | 4.00 x 10^6^ | [[p 1071 in 14](#_ENREF_14)] |
| Rat 1-year drinking water study | 365 | Males **37.8** mg kg bw⁻¹ day⁻¹ ^a^ | 3.15 x 10^5^ | 1.06 x 10^6^ | UBA |
| ^a^ Dekant and Dekant [[14](#_ENREF_14)] point out that the German UBA (Umweltbundesamt), which commissioned the study, considered the small increases in the activity of alanine transaminase (an enzyme found mainly in the liver) observed at 1.8 mg kg bw⁻¹ day⁻¹ were more appropriate for estimation of the NOEL, and used this to derive a guideline for TFA in drinking water. Hypertrophy of the liver and increased expression of enzymes in the liver is commonly observed in toxicity tests in rodents but is usually reversible when exposure ceases [[18](#_ENREF_18)]. A recovery period was not included in the study.  The bolded numbers are the NOELs most suitable for assessing risks to humans. | | | | | |

The teratology (developmental) study in female rabbits [[reported in 17](#_ENREF_17)] is summarised here. Briefly, the study followed OECD TG414 and the Na-TFA was 99.9% pure and was administered in an aqueous solution. Concentrations were verified by chemical analysis of the dosing solutions. The rabbits were mated and acclimated until gestation day-5 and administered by gavage with 10 ml kg bwˉ¹ from day 6–28. There were three dose-groups with 24 females per group and the doses were 180, 375, and 750 mg kg bw⁻¹ day⁻¹ and were based on a range-finding test. On day-29 the study was terminated, and necropsy of the females showed a dose-related increase in weight of the liver relative to body weight., which was accompanied by fibrosis. The maternal NOEL was 180 mg kg bw⁻¹ day⁻¹. Dose-related foetal abnormalities such as major eye abnormalities, multiple folded retinas and absent aqueous/vitreous humour were observed in the foetuses from the dams treated with 375 and 750 mg kg bw⁻¹ day⁻¹. The incidence of these abnormalities was significantly greater than in the concurrent and historical controls (HCD). Unfortunately, the ECHA summary did not provide raw data, such as the total number of foetuses in each dose group. References were made to “attached Tables” but these were not accessible in the ECHA summaries. One foetus in the 180 mg kg bw⁻¹ day⁻¹ had similar abnormalities of the eye to those in the higher doses, so this is the Lowest Observed Effect Level (LOEL). Because doses < 180 mg kg bw⁻¹ day⁻¹ were not included, a NOEL could not be determined. Thus, these data were not usable in risk assessment.

**References**

1. Madronich, S., Sulzberger, B., Longstreth, J. D., Schikowski, T., Andersen, M. P. S., Solomon, K. R., et al. (2023). Changes in tropospheric air quality related to the protection of stratospheric ozone in a changing climate. *Photochemical & Photobiological Sciences, 22*, 1129–1176, <https://doi.org/10.1007/s43630-023-00369-6>.

2. Saez, C., Bautista, A., Nikolenko, O., Scheiber, L., Llorca, M., Jurado, A., et al. (2024). Occurrence and fate of perfluoroalkyl and polyfluoroalkyl substances (PFAS) in an urban aquifer located at the Besos River Delta (Spain). *Environmental Pollution, 358*, 124468, <https://doi.org/10.1016/j.envpol.2024.124468>.

3. Wang, Y., Liu, L., Qiao, X., Sun, M., Guo, J., Zhao, B., et al. (2024). Atmospheric fate and impacts of HFO-1234yf from mobile air conditioners in East Asia. *Science of the Total Environment, 916*, 170137, <https://doi.org/10.1016/j.scitotenv.2024.170137>.

4. Liang, S.-H., Steimling, J. A., & Chang, M. (2023). Analysis of ultrashort-chain and short-chain (C1 to C4) per-and polyfluorinated substances in potable and non-potable waters. *Journal of Chromatography Open, 4*, 100098, <https://doi.org/10.1016/j.jcoa.2023.100098>.

5. Albers, C. N., & Sültenfuss, J. (2024). A 60-year increase in the ultrashort-chain PFAS trifluoroacetate and its suitability as a tracer for groundwater age. *Environmental Science & Technology Letters, 11*, 1090-1095, <https://doi.org/10.1021/acs.estlett.4c00525>.

6. RIWA (2018). Annual Report 2017 – The Rhine. pp. 131. <https://www.riwa-rijn.org/publicatie/jaarrapport-2018-de-rijn/>.

7. RIWA (2019). Annual Report 2018 – The Rhine pp. 209. <https://www.riwa-rijn.org/publicatie/jaarrapport-2020-de-rijn/>.

8. RIWA (2020). Annual Report 2019 – The Rhine. pp. 168. <https://www.riwa-rijn.org/publicatie/jaarrapport-2020-de-rijn/>.

9. RIWA (2021). Annual Report 2020 – The Rhine. pp. 141. <https://www.riwa-rijn.org/publicatie/jaarrapport-2021-de-rijn/>.

10. RIWA (2022). Annual Report 2021 – The Rhine. pp. 137. <https://www.riwa-rijn.org/publicatie/jaarrapport-2022-de-rijn/>.

11. RIWA (2023). Annual Report 2022 – The Rhine. pp. 141. <https://www.riwa-rijn.org/publicatie/jaarrapport-2023-de-rijn/>.

12. RIWA (2024). Annual Report 2023 – The Rhine. pp. 147. <https://www.riwa-rijn.org/publicatie/jaarrapport-2023-de-rijn/>.

13. Eurofins (2023). Ultrashort PFAS in Swedish and Norwegian Drinking Water. pp. 7. <https://www.eurofins.se/tjaenster/miljoe-och-vatten/nyheter-miljo/eurofins-study-ultrashort-pfas-in-swedish-and-norwegian-drinking-water/>.

14. Dekant, W., & Dekant, R. (2023). Mammalian toxicity of trifluoroacetate and assessment of human health risks due to environmental exposures. *Archives of Toxicology, 97*(4), 1069-1077, <https://doi.org/10.1007/s00204-023-03454-y>.

15. US EPA (2023). ORD Human Health Toxicity Value for Perfluoropropanoic Acid. pp. 58.

16. US EPA (2022). IRIS Toxicological Review of Perfluorobutanoic Acid (PFBA, CASRN 375-22-4) and Related Salts. pp. 145.

17. ECHA (2024). Trifluoracetic Acid, Rabbit Teratogenicity Study. <https://chem.echa.europa.eu/100.018.982/dossier-view/b54eeda3-2d2f-4376-b28b-aa14ed94edcb/f300bb5a-ed46-4f45-9141-ca60d27abfb0_f300bb5a-ed46-4f45-9141-ca60d27abfb0?searchText=TFA>. Accessed September 2024.

18. Felter, S., Foreman, J., Boobis, A., Corton, J., Doi, A., Flowers, L., et al. (2018). Human relevance of rodent liver tumors: Key insights from a Toxicology Forum workshop on nongenotoxic modes of action. *Regulatory Toxicology and Pharmacology, 92*, 1–7, <https://doi.org/10.1016/j.yrtph.2017.11.003>.
